# Reprogramming of fatty acid metabolism: a hidden force regulating the occurrence and progression of cholangiocarcinoma

**DOI:** 10.1038/s41420-025-02351-w

**Published:** 2025-02-21

**Authors:** Jinglei Zhang, Kaiyi Ruan, Zhuohuan Chu, Xiang Wang, Ye Gu, Hangbin Jin, Xiaofeng Zhang, Qiang Liu, Jianfeng Yang

**Affiliations:** 1 The Fourth School of Clinical Medicine, Zhejiang Chinese Medical University, Hangzhou First People’s Hospital, Hangzhou, Zhejiang Province 310053 China; 2 Department of Gastroenterology, Affiliated Hangzhou First People’s Hospital, Westlake University School of Medicine, Hangzhou, Zhejiang Province 310006 China; 3 Zhejiang University School of Medicine, Hangzhou, Zhejiang Province 310058 China; 4 Key Laboratory of Integrated Traditional Chinese and Western Medicine for Biliary and Pancreatic Diseases of Zhejiang Province, Hangzhou, Zhejiang Province 310006 China; 5 Hangzhou Institute of Digestive Diseases, Hangzhou, Zhejiang Province 310006 China

**Keywords:** Fatty acids, Bile duct cancer

## Abstract

Cholangiocarcinoma (CCA) is a malignant tumor that originates from the bile duct epithelium and with a poor outcome due to lack of effective early diagnostic methods. Surgical resection is the preferred method for cure, but treatment options are limited for advanced diseases, such as distant metastatic or locally progressive tumors. Therefore, it is urgent to explore other new treatment methods. As modern living standards rise, the acceptance of high-fat, high-protein, and high-carbohydrate diets is growing among the public, and the resulting metabolic abnormalities are intimately linked to the initiation and spread of tumors. Metabolic reprogramming is a key mechanism in the process of tumor development and progression and is closely related to cancer cell proliferation, metastasis and drug resistance. Fatty acid (FA) metabolism, an integral component of cancer cell metabolism, can provide an energy source for cancer cells and participate in cell signaling, the regulation of the immune response and the maintenance of homeostasis of the internal environment, which are closely linked to the development and progression of CCA. Therefore, a better understanding of FA metabolism may provide promising strategies for early diagnosis, prognostic assessment and targeted therapy for CCA patients. In this paper, we review the effects of FA metabolism on CCA development and progression, summarize related mechanisms and the existing clinical applications of targeted lipid metabolism in CCA, and explore new targets for CCA metabolic therapy.

## FACTS

- Compared with normal bile duct epithelial cells, CCA cells meet the need for tumor cell deterioration by enhancing FA metabolism. FAs are maintained mainly through two pathways: exogenous intake and endogenous synthesis.
- Acetyl-CoA carboxylase and fatty acid synthase are key enzymes that drive FA de novo synthesis. When the expression level of fatty acid synthase is low, CCA cells provide FAs for cancer development and progression by increasing the expression of FA uptake-related proteins.
- In an environment of high levels of oxidative damage, bile duct epithelial cells can undergo malignant transformation to CCA. Fatty acid oxidation is the preferred energy source for highly proliferating CCA cells.
- The core of ferroptosis is the lipid peroxidation of highly expressed unsaturated fatty acids in the cell membrane in response to ferrous ions or lipoxygenase. Ferroptosis is closely associated with the malignant progression, drug resistance and poor prognosis of CCA.
- ω-3 polyunsaturated fatty acids mainly exert anti-oncogenic effects, while ω-6 polyunsaturated fatty acids mainly have pro-oncogenic effects, and saturated fatty acids have different mechanisms of action on CCA according to their class. FAs can synthesize other lipids to participate in tumor metabolic reprogramming.

## Background

CCA is an epithelial malignancy originating in the biliary tree. It is the second most common primary liver cancer after hepatocellular carcinoma (HCC) and is typically classified into three subtypes based on anatomical structure: intrahepatic CCA (iCCA), perihilar CCA (pCCA) and distal CCA (dCCA) [1, 2]. Due to the absence of early specific clinical manifestations and precise diagnostic methods, most patients are diagnosed at advanced stages [3]. The incidence and mortality of CCA have increased over the last few decades, accounting for approximately 3% of gastrointestinal malignancies and 15% of primary liver cancers [4]. At present, surgery is the preferred treatment option. However, more than 60% of patients experience recurrence and metastasis after surgery, and the 5-year survival rate is only 25%-50% [5, 6]. Moreover, although combination therapy of gemcitabine (GEM) with cisplatin is a standard first-line therapy, the effect of adjuvant chemotherapy in patients with regional lymph node metastasis, positive margins and low differentiation is not significant, and the overall survival time is < 1 year [7]. Therefore, the early diagnosis and treatment of CCA is of great medical and social significance. With improvements in modern living standards, people are becoming increasingly prone to eating meals that are heavy in protein, fat and carbohydrates. However, this can lead to metabolic disorders and a host of other ailments, including cancers, obesity and inflammation. In recent years, changes in cell metabolism that contribute to tumorigenicity and cancer progression have attracted the interest of researchers. The metabolic reprogramming of cancer cells refers to the phenomenon of cancer cells adjusting and changing their metabolic pathways during development, which is the eighth major feature of cancer cells after self-activation of growth signals, insensitivity to growth inhibition signals, escape from apoptosis, unlimited replication ability, continuous angiogenesis, and tissue invasion and metastasis [8]. These alterations mainly include glucose metabolism, glutamine metabolism, and lipid metabolism. As an important part of metabolic reprogramming, lipid metabolism is involved in the composition of biological membranes and structural units, energy storage, and plays a role as a signaling pathway in a variety of cellular activities. At present, extensive evidence has revealed that lipid metabolism reprogramming is inseparable from tumorigenesis and development. FAs, as basic components of lipids, are widely involved in the occurrence and development of CCA, including its synthesis, transport and degradation. Furthermore, FAs are closely related to CCA treatment and drug resistance. Therefore, targeted FA metabolism therapy has gradually become an important method for cancer treatment and drug resistance treatment. In this review, we summarize how FA metabolic reprogramming contributes to the development and progression of CCA and discuss therapeutic strategies for targeting FA metabolism in CCA.

## Fa De Novo Synthesis Provides A Source Of Lipids For Cca Genesis And Development

**Fig. 1:**
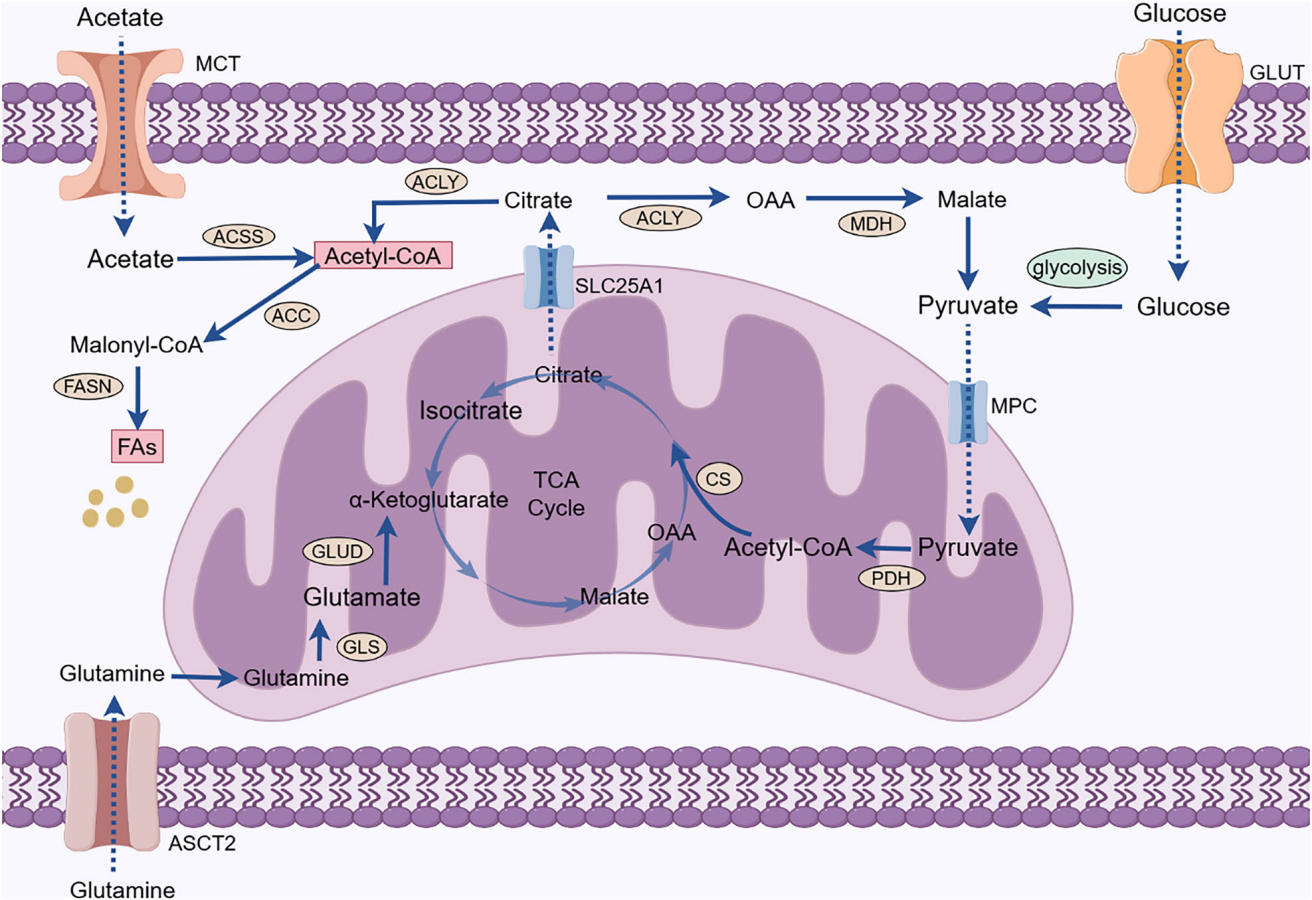
The sources of acetyl-CoA and FA de novo synthesis in cancer cells. Acetyl-CoA is a crucial component in FA de novo synthesis and is obtained by the metabolism of glucose, glutamine, and acetate. Pyruvate is the end product of glycolysis and is transported to the mitochondria via MPC to participate in the TCA cycle to produce citrate. Glutamine enters cancer cells via ASCT2 and is converted in the mitochondria to glutamate by a deamination reaction. Glutamate is converted by glutamate dehydrogenase to α-ketoglutarate, which is an intermediate product of the TCA cycle that further produces citrate. Citrate in mitochondria enters the cytoplasm with the assistance of SLC25A1. Citrate can be catalyzed by ACLY to generate acetyl-CoA. Moreover, ACSS catalyzes the activation of acetate to yield acetyl-CoA. Acetyl-CoA is used in lipid synthesis to generate FAs catalyzed by ACC and FASN. (GLUT, glucose transporter; MPC, mitochondrial pyruvate carrier; PDH, pyruvate dehydrogenase; CS, citrate (Si)-synthase; OAA, oxaloacetic acid; TCA, tricarboxylic acid; GLUD, glutamate dehydrogenase; GLS, glutaminase; ASCT2, alanine-serine-cysteine transporter 2; ACLY, ATP-citrate lyase; MDH, malate dehydrogenase; ACC, acetyl-CoA carboxylase; FASN, fatty acid synthase; ACSS, acetyl-CoA synthetase; SLC25A1, solute carrier family 25 member 1; FAs, fatty acids).

Mammalian FAs are mainly derived from two pathways: exogenous uptake from the surrounding microenvironment and endogenous synthesis via the use of nutrients. FA endogenous synthesis, also known as FA de novo synthesis, is carried out in the cytoplasm using acetyl-coenzyme A (acetyl-CoA), adenosine 5’-triphosphate (ATP), and biotin as raw materials. Under physiological conditions, normal cells meet lipid requirements primarily through the direct uptake of exogenous FAs, and lipogenesis is primarily restricted to hepatocytes and adipocytes. However, cancer cells can activate lipogenesis to meet the needs of rapid proliferation [9]. Acetyl-CoA is a crucial component in FA de novo synthesis and is obtained by the metabolism of glucose, glutamine and acetate. Glucose-derived pyruvate is catalyzed by pyruvate dehydrogenase in mitochondria to form acetyl-CoA, which is then condensed with oxaloacetate to form citrate by citrate synthase. Citrate not only can complete the tricarboxylic acid (TCA) cycle in mitochondria but can also be carried to the cytoplasm by the citrate transporter SLC25A1 and converted to acetyl-CoA by ATP-citrate lyase (ACLY). Additionally, glutamine contributes to citrate production through reductive carboxylation. In addition, under conditions of metabolic stress such as hypoxia or lipid depletion, cancer cells can accelerate acetate activation to generate acetyl-CoA by upregulating acetyl-CoA synthetase (ACSS) expression. Eventually, acetyl-CoA is first synthesized into palmitic acid (PA) with 16-carbon atoms by key enzymes, such as acetyl-CoA carboxylase (ACC) and fatty acid synthase (FASN), and then converted to other FAs by altering the length of the carbon chain or adding unsaturated bonds. FA de novo synthesis is beneficial for increasing the saturation of cellular and organelle membranes, preventing death by counteracting lipid peroxidation (LPO) and attenuating drug uptake by changing transverse membrane dynamics [10]. Therefore, FA de novo synthesis is highly important for cancer occurrence and progression. Acetyl-CoA and various enzymes mediating FA synthesis play key roles in FA anabolism (Fig.1).

ACC is the main regulator of FA metabolism and occurs in two isoforms: ACC1 and ACC2. ACC1 is located in the cytoplasm. It is a rate-limiting enzyme for FA de novo synthesis and can catalyze the carboxylation of acetyl-CoA to malonyl-CoA. However, ACC2 is anchored to the outer mitochondrial membrane. It can regulate FA uptake and reduce fatty acid oxidation (FAO) by producing products that inhibit carnitine palmitoyltransferase-1 (CPT-1) [11]. Therefore, ACC is critical for FA metabolism. Previous research has shown that ACC1 was highly expressed in various human cancers, including HCC, gastric cancer and lung cancer. ACC1 depletion attenuates FA de novo synthesis and inhibits cancer development [12–14]. Saisomboon S et al. [15] found that ACC1 could affect the invasion and migration ability of CCA by regulating the AMPK-NF-κB-Snail axis, and for CCA patients, higher ACC1 expression often indicated shorter survival. AMP-activated protein kinase (AMPK) is the regulatory center of energy metabolism and is widely involved in the regulation of numerous enzymes and transcription factors associated with lipid metabolism [16]. ACC has been shown to be a target molecule of AMPK, and AMPK phosphorylation can regulate ACC activity. Under low-energy conditions, AMPK can increase ATP content, attenuate ACC activity and inhibit lipid anabolism by phosphorylating specific enzymes and sites [17]. Similarly, berberine suppresses tumor growth by blocking ACC activity through the activation of AMPK, leading to a reduction in intracellular FA synthesis and extracellular vesicle production [18]. Following the downregulation of ACC1 expression in CCA, there is a steady decrease in the synthesis of malonyl-CoA and PA, which leads to a decrease in the amount of neutral lipids and ATP and an increase in AMPK phosphorylation. An increase in p-AMPK can reduce the Snail-mediated invasion and migration of cancer cells by inhibiting the nuclear translocation of NF-κB p65, ultimately inhibiting the occurrence and development of CCA. 5-tetradecyloxy-2-furoic acid (TOFA) has been shown to have cytotoxic effects as an ACC metastable inhibitor in a variety of tumors, including lung, colon, and renal cancers. Boonnate P et al. [19] reported that TOFA attenuated the endogenous synthetic capacity of FAs by downregulating ACC expression and inhibited CCA cell proliferation in a dose-dependent manner. In summary, these findings suggest that ACC plays an important role in CCA and that targeting ACC may improve the poor prognosis of CCA patients by regulating de novo FA synthesis.

**Fig. 2:**
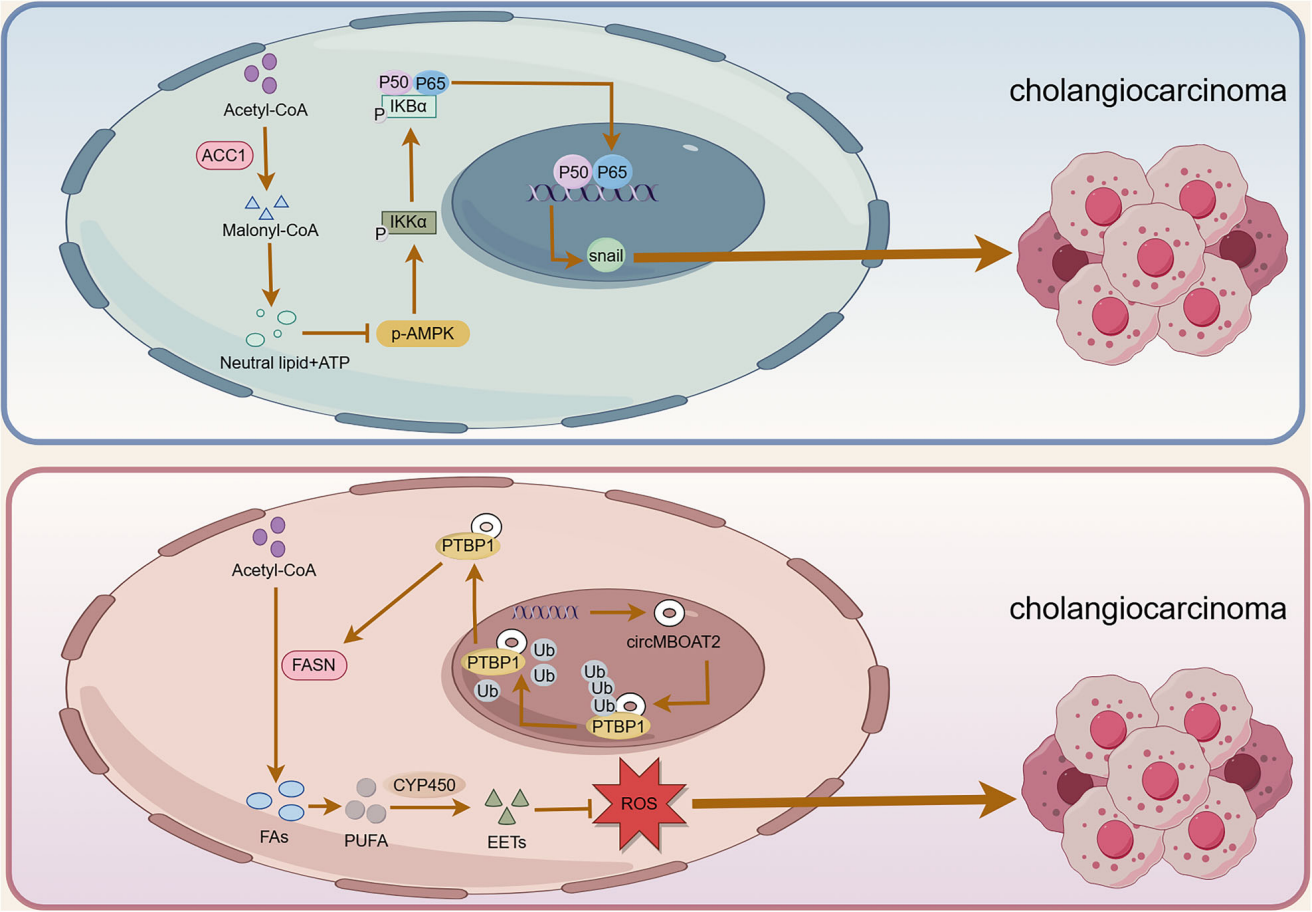
Impact of ACC and FASN on CCA occurrence and progression. ACC and FASN, key enzymes in FA de novo synthesis, regulate the development and progression of CCA. Increased ACC1 expression in CCA can accelerate the accumulation of neutral lipids and ATP, which in turn inhibit the phosphorylation of AMPK. Reduced p-AMPK accumulation can ultimately accelerate the development and progression of CCA by enhancing the nuclear translocation of NF-κB p65. Moreover, circMBOAT2, the expression of which is upregulated in iCCA, protects PTBP1 from ubiquitin/proteasome-dependent degradation. PTBP1 accelerates the cytoplasmic export of FASN mRNA, which drives the generation of PUFAs. PUFAs produced by circMBOAT2 are mainly catalyzed by the CYP450 pathway to produce EET, which has anti-oxidative and antiapoptotic effects. Ultimately, PUFAs and EET inhibit lipid peroxidation and ROS level, and promote the malignant tumor progression. (ACC1, acetyl-CoA carboxylase 1; ATP, adenosine 5’-triphosphate; AMPK, AMP-activated protein kinase; IKKα, inhibitor of kappa B kinase α; FASN, fatty acid synthase; FAs, fatty acids; PUFAs, polyunsaturated fatty acid; PTBP1, polypyrimidine tract-binding protein 1; Ub, ubiquitin; ROS, reactive oxygen species; CYP450, cytochrome P450; EET, epoxyeicosatrienoic acid).

In addition, FASN, a key lipogenic enzyme in FA de novo synthesis, condenses one molecule of acetyl-CoA and seven malonyl-CoA molecules into PA, which is used for the synthesis of more complex FAs, plasma membrane structures, and posttranslational protein palmitoylation [20]. FASN has been widely studied in a variety of cancers. Its overexpression is associated with advanced tumor stage, lymph node metastasis, distant metastasis, and short survival time [21, 22]. FASN contributes to the occurrence and progression of CCA through various mechanisms. Tomacha J et al. [23] proposed that FASN overexpression was closely related to the proliferation, migration and invasion of CCA cells, as well as the poor prognosis and high recurrence risk of patients, and can be used as a biomarker to predict the invasiveness and prognosis of CCA. In a database of 155 CCA patients, there was a clear correlation between the advanced state of CCA patients and the expression of FASN. In other words, CCA patients with higher FASN expression have a worse prognosis. Moreover, when FASN was knocked down, the growth, migration and invasion of CCA cells were inhibited, the cell cycle was arrested, and apoptosis was induced. Metabolomic analysis revealed that the expression of guanine and xanthine was significantly greater in the FASN knockdown group than in the control group. As the most abundant metabolite in living organisms, purine is a key component of DNA, RNA and basic biomolecules. To meet their needs for rapid proliferation, cancer cells often exhibit increased purine production in comparison to that of normal cells [24]. In CCA, the inhibition of FASN expression hinders purine metabolism, which in turn attenuates ATP and DNA production, leading to the inhibition of CCA cell proliferation and the induction of apoptosis. Circular RNAs (circRNAs), whose biogenesis is regulated by specific cis-acting elements and trans-acting factors, are covalently closed, endogenous biomolecules in eukaryotes with tissue-specific and cell-specific expression patterns. circRNAs have been shown to be dysregulated in a variety of cancers and have emerged as potential biomarkers and therapeutic targets [25]. CircMBOAT2 is a circRNA derived from MBOAT2 that is closely related to lipid metabolism. Yu X et al. [26] reported that circMBOAT2 is positively correlated with the malignant features of iCCA. The results confirmed that circMBOAT2, the expression of which is upregulated in iCCA, protected polypyrimidine tract-binding protein 1 (PTBP1) from ubiquitin/proteasome-dependent degradation by binding to PTBP1, which in turn facilitated PTBP1-mediated cytoplasmic export of FASN mRNA, driving polyunsaturated fatty acids (PUFAs) and oxidized lipids production. Oxidized lipids have an important regulatory role in the initiation, development and regression of inflammation. Oxidized lipids also have a role in regulating intracellular redox state, in which some oxylipins promote the production of oxidants or are highly active oxidants themselves, while others have the ability to inhibit the production of pro-oxidants or promote the production of antioxidants [27]. The researchers speculate that the PUFAs produced by circMBOAT2 are mainly catalyzed by the cytochrome P450(CYP450) pathway to produce epoxyeicosatrienoic acid (EET), which has anti-oxidative and antiapoptotic effects on endothelial cells and other cells. Ultimately, PUFAs and EET inhibit lipid peroxidation and ROS level, and promote the malignant tumor progression. This suggested that silencing circMBOAT2 could be a novel strategy for treating iCCA, especially for patients with active lipid metabolism. In addition, Zhang B et al. [28] reported that KDM5C, which is expressed at low levels in iCCA and closely related to FASN expression, may exert oncogenic effects as a novel class of tumor suppressors. KDM5C is reported to be a histone H3K4-specific demethylase that is associated with tumor progression and poor prognosis [29, 30]. In iCCA, the overexpression of KDM5C hinders the synthesis of FAs by attenuating the modification of H3K4me3 on the promoter of the FASN gene and subsequently decreasing the transcriptional activation of FASN mRNA, impeding the proliferation, migration and invasion of CCA cells. KDM5C is involved in the pathogenesis of iCCA by targeting FASN, and it could be a potential therapeutic target for iCCA in the future. Moreover, spindle and kinetochore-associated complex subunit 3 (SKA3) can also be used as a potential biomarker to monitor the prognosis of CCA patients. SKA3 is closely related to tumor development. When SKA3 expression is reduced, cells induce mid-mitotic arrest by activating the spindle component checkpoint and reducing sister chromatid cohesion. Chen Y et al. [31] reported that hypoxia-induced SKA3 promoted CCA cell proliferation and increased GEM chemoresistance through the upregulation of FASN expression in ex vivo experiments. Under hypoxic conditions, SKA3 can bind to HIF-1α by recruiting PARP1, which in turn enhances the poly ADP-ribosylation of HIF-1α, induces the USP7-mediated deubiquitination of HIF-1α, and ultimately upregulates key lipogenic enzymes to increase the level of lipid expression, which provides a source of energy to drive the malignant proliferation of tumor cells. Currently, TVB-2640, a second-generation targeted drug for FASN, has entered the phase 2 clinical trial stage and has been confirmed to have safe and effective cancer inhibitory effects [32]. Although there are few clinical studies on FASN inhibitors for the treatment of CCA, FASN is closely related to CCA, and the efficacy and safety of targeting FASN for the treatment of patients with CCA should be investigated. In summary, the levels of ACC and FASN, key enzymes involved in FA de novo synthesis, are closely related to the development and progression of CCA (Fig. 2). Therefore, the regulation of FA de novo synthesis plays a critical role in combating CCA.

## Cca Enhances Fa Uptake Via Multiple Transport Mediators To Accelerate Tumor Malignancy

**Fig. 3:**
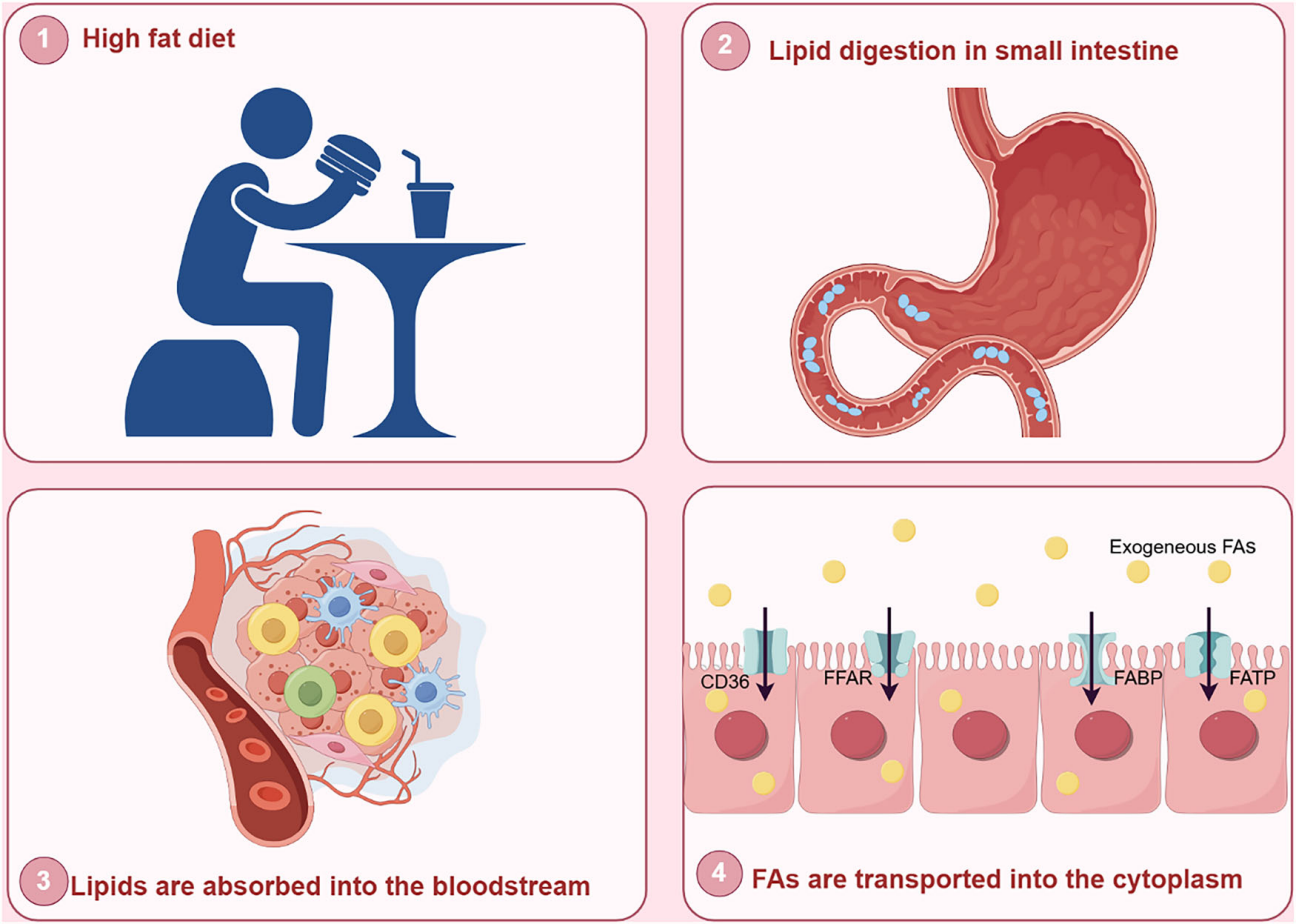
Process of lipid uptake. A high-fat diet accelerates the body’s lipid intake. Lipids are first digested into small molecules in the stomach and intestines and then absorbed into the blood, where they can participate in the formation of the tumor microenvironment. The exogenous uptake of FAs from the surrounding microenvironment is facilitated by specialized transporters, including CD36, FATP, FABP and FFAR. (CD36, fatty acid translocase; FATP, fatty acid transport protein; FABP, fatty acid-binding protein; FFAR, free fatty acid receptor; FAs, fatty acids).

CCA is a heterogeneous group of malignancies based on its histological and molecular features [33]. It may emerge at different sites in the biliary tree and has different macroscopic or morphological characteristics [34]. Therefore, not all CCAs rely on FA de novo synthesis as a source of bioenergetics, and the enhanced exogenous uptake of FAs can also contribute to the progression and survival of CCAs. A related report indicated that some oncogene-induced iCCA cells inhibited FA de novo synthesis and exhibited strong exogenous uptake [35]. The ablation of FASN in a mouse model and in vitro livers did not affect AKT/NCID-induced iCCA formation, and CCA cells with low expression levels of FASN tended to have high expression levels of FA uptake-associated proteins and robust FA uptake capacity [36, 37]. This indicates that iCCA cells may undergo membrane formation in the absence of FASN by boosting the absorption of supplemental lipids from exogenous FAs to facilitate tumor progression. It is evident that cancer cells possess the capacity to regulate the source of FAs and that different types of CCA cells are capable of selecting between endogenous synthesis and exogenous uptake of FAs to fulfill their lipid requirements, contingent on their specific circumstances. Thus, FA de novo synthesis and exogenous uptake complement each other and play important roles in maintaining FA homeostasis in the body. In general, a high-fat diet could accelerate the body’s lipid intake. Exogenous uptake of lipids usually contains triglycerides, which can be directly ingested by intestinal mucosal cells after being emulsified by bile acids, and then hydrolyzed into FAs and glycerol under the action of intracellular lipase, which enter the blood circulation through the portal vein [38]. However, due to the hydrophobic nature of FAs, exogenous FAs frequently require a transport mediator to enter the cell and exert their effects(Fig. 3). As crucial transport mediators, cluster of differentiation 36 (CD36), fatty acid transport proteins (FATPs), fatty acid binding proteins (FABPs) and free fatty acid receptors (FFARs) have been shown to be highly expressed in CCA and participate in tumorigenesis and progression through a multitude of mechanisms(Table 1).

**Table. 1:** The role of FA transport mediators in CCA.

Transport mediator		Type of CCA	Brochure	Research	Ref.
CD36		CCA	91 serum from CCA patients	When CD36 expression levels are elevated, lipid uptake is increased, and the increased lipid content in turn facilitates the survival of CSCs in the TME, ultimately increasing the risk of recurrence in patients.	[47]
FATPs	FATP1	iCCA	70 samples of iCCA and corresponding non-tumorous surrounding livers	The upregulation of FATP1 expression leads to a greater likelihood of deterioration of iCCA.	[36]
FABPs	FABP1	CCA	17071 samples from 150 different tumor types	The positive rate of FABP1 in CCA is only 21.6%.	[53]
		HCCA	132 patients with HCCA	The expression of FABP1 is elevated in HCCA.	[54]
	FABP4	CCA	CCA cells (RBE and HCCC-9810)	FABP4 mediates the adipocyte-induced invasion, migration and EMT of CCA cells.	[59]
	FABP5	iCCA	16 patients with IMCC	In IMCC, FABP5 expression is elevated and is positively correlated with tumor size, lymph node metastasis, vascular infiltration and TNM stage.	[62]
		BTC	74 surgical resection specimens	The expression levels of FABP5 in large BTC (periportal CCA, eCCA and gallbladder cancer) are greater than those in small BTC (iCCA and cholangiocellular carcinoma) and that the prognosis of patients is worse.	[63]
FFARs	FFAR4	CCA	98 samples of CCA tissues and adjacent tissues	High FFAR4 expression implies an increase in the EMT capacity of CCA cells and a decrease in the disease-free survival and overall survival of CCA patients.	[68]

### CD36

CD36 is a B2 receptor of the scavenger receptor class B family and is composed of single-chain secondary transmembrane glycoproteins; it is expressed in cancer cells, stromal cells and immune cells, but its expression varies among distinct cell types and tumor stages. CD36 widely participates in biological processes, such as immunomodulation and metabolic regulation. In lipid metabolism, CD36 mainly acts as a receptor protein for long-chain fatty acids (LCFAs). Benton CR et al. [39] reported that, compared with that in wild-type mice, the uptake rate of LCFAs in skeletal muscle was significantly decreased in mice with CD36 knocked out and that the uptake rate of LCFAs could be restored to normal when CD36 was retransferred into defective mice. These findings suggested that CD36 was an indispensable transporter and mediator of LCFA uptake and absorption. According to a recent study, CD36 may be able to control the transport of FAs by utilizing dynamic palmitoylation-regulated endocytosis [40]. Palmitoylation refers to the binding of PA to amino acid residues of proteins through thioester bonds; it is a dynamic and reversible form of posttranslational modification that occurs widely in organisms and is important for regulating protein transport, cellular localization and stability. The isoforms of aspartate-histidine-histidine-cysteine (DHHC), such as DHHC4 and DHHC5, can dynamically palmitoylate CD36 at the Golgi and cytoplasmic membranes to maintain the plasma membrane localization of CD36 and enhance the cellular uptake of FAs. Blocking CD36 palmitoylation or inhibiting its secretion is important for diminishing FA uptake by cancer cells. It is now widely accepted that CD36 is overexpressed in a large variety of tumors and is involved in metastasis initiation, the proliferation of metastatic cells and tumor drug resistance [41–43]. For example, aggressive breast cancer cells exhibit significantly lower levels of CD36 than aggressive breast cancer cells [44]. Additionally, a study demonstrated that tumor-associated adipocytes could enhance the ability of breast cancer cells to take up FAs and enhance tumor invasiveness by secreting CD36 [45]. In parallel, CD36 expression is upregulated in lapatinib-resistant breast cancer cells, and targeting CD36 restores sensitivity to lapatinib [46]. With respect to the relationships among CCA, FAs and CD36, researchers have found that patients who experienced CCA recurrence tended to have decreased levels of metabolites involved in mitochondrial respiration and increased lipid uptake compared to nonrecurrent patients [47]. This metabolic signature is closely tied to both CD36 and cancer stem cells (CSCs). CSCs and CD36 are interconnected factors that contribute to CCA recurrence and are positively correlated. In the case of CCA, when CD36 expression levels are elevated, lipid uptake is subsequently increased, and the increased lipid content in turn facilitates the survival of CSCs in the tumor microenvironment (TME), ultimately increasing the risk of recurrence in patients. Consequently, the lower the level of lipids in the body is, the lower the rate of recurrence in patients with CCA. In addition, CD36 can also induce tumor immune tolerance and progression by driving lipid metabolic reprogramming in tumor-associated immune cells [48]. CD36 is capable of maintaining the mitochondrial function of regulatory T cells (Tregs) in the TME through the peroxisome proliferator-activated receptor (PPAR) signaling pathway. For instance, in lung cancer and melanoma, increasing CD36 expression on Tregs can enhance the survival of Tregs in a lactate-rich TME via the activation of the PPAR-β signaling pathway and augmentation of the immunosuppressive properties of the TME [49]. In contrast, strong expression of CD36 is associated with lipid oxidation accumulation and ferroptosis in CD8^+^ T cells. Increased CD36 attenuates the ability of CD8^+^ T cells to release cytotoxic factors and promote tumor progression [50]. In conclusion, CD36 is important for the occurrence, development and prognosis of CCA. Targeting CD36-mediated FA endocytosis not only serves as an invaluable method for preventing CCA recurrence but also as an effective strategy to increase the efficacy of tumor immunotherapy.

### FATPs

FATPs are members of the solute carrier 27 (SLC27) family due to their role in the transfer of external FAs. These proteins are localized at the plasma membrane or at the junction of the intracellular membrane and the endoplasmic reticulum and act as gates in regulating the transport of FAs [51]. Therefore, they are therapeutic targets. As key factors involved in FA transport and fat deposition, FATPs can assist in the transmembrane transport of LCFAs and have acyl-CoA synthetase activity. Related reports have indicated that FATPs could be involved in the progression of various tumors, such as melanoma, breast cancer, prostate cancer, renal cell carcinoma and HCC, through the regulation of lipid metabolism [52]. FATP1 plays a crucial role as a FA transporter in CCA cells. In their investigation of the relationship between FATP1 and CCA, Li L et al. [36] reported that the upregulation of FATP1 expression in iCCA cells led to an increase in the efficiency of FA de novo synthesis, resulting in a greater likelihood of deterioration of iCCA. Thus, high FATP1 expression can promote the progression and development of CCA, and targeting FABP1 may provide a new target for the prevention and treatment of CCA. However, there are few studies on the correlation between FATPs and CCA, and it is challenging but necessary to further explore the relevant mechanism to clarify this association.

### FABPs

FABPs belong to the intracellular lipid-binding protein superfamily and consists of four exons and three introns. At least nine FABP isoforms have been identified. As lipid chaperone proteins, FABPs participate in LCFA transport and metabolic regulation by binding hydrophobic lipid ligands and play an instrumental role in energy storage, signaling, and immune regulation. To address the role of FABPs in the development and progression of CCA, existing studies have shown that FABP1, FABP4 and FABP5 are closely related to CCA. FABP1, also known as hepatic FABP, is most abundantly expressed in the liver, accounting for approximately 10% of the total cytoplasmic proteins. It is of diagnostic utility for tumors. After comprehensively determining the expression of FABP1 in normal and tumor tissues, Dum D et al. [53] found that FABP1 expression was highly tumor specific, with the highest positive rates in colorectal adenomas (86%), colorectal adenocarcinomas (71.1%) and HCC (65.3%), followed by ovarian mucinous carcinoma (34.6%) and various gastrointestinal adenocarcinomas (10–23%); the positive rate of FABP1 in CCA is only 21.6%. Moreover, compared with that in other types of CCA, the expression of FABP1 was significantly elevated in hilar cholangiocarcinoma (HCCA). A reduced survival rate and increased tumor size are linked to low FABP1 expression. This finding is highly important for the diagnosis of HCCA, and the FABP1 expression level is also considered an independent prognostic factor for HCCA patients after radical resection [54]. However, although there is some correlation between FABP1 and the diagnosis and prognosis of CCA, FABP4 and FABP5 are more closely associated with CCA. FABP4, also known as adipose-FABP (A-FABP), is predominantly derived from adipocytes and can enhance lipid transport capacity and activate multiple oncogenic signaling pathways through multiple pathways. Therefore, FABP4 is a key driver of tumorigenesis. The upregulation of FABP4 expression in the TME not only promotes lipid droplet formation and attenuates oxidative stress and ferroptosis but also induces epithelial–mesenchymal transition (EMT) [55, 56]. Tumors near the greater omentum and mesentery are often surrounded by a lipid-rich tumor microenvironment due to the presence of adipose tissue in these areas, which is a major component of visceral fat. Adipocytes and FABP4 work together extensively to regulate pathological processes such as tumor stromal remodeling and EMT [57]. FABP4 is overexpressed in ovarian metastatic carcinoma of the greater omentum, and the inhibition of FABP4 results in limited ovarian cancer metastasis [58]. Nie J et al. [59] was the first to demonstrated the criticality of FABP4 in the interaction between adipocytes and CCA cells. When CCA cells were cocultured with adipocytes and their extracts, FABP4, MMP-2, MMP-9 and snail expression was upregulated. EMT in tumor cells was correspondingly attenuated after FABP4 expression was suppressed. Therefore, the development of CCA is closely related to FABP4. In addition, FABP5 also participates in bile duct cell malignancy. The Japanese Hepatocellular Carcinoma Study Group classified iCCA into mass type, periductal infiltrating type, and intrahepatic growing type based on morphological features. The etiology, risk factors, prognosis and treatments of the three types are different, among which mass-type intrahepatic mass-forming cholangiocarcinoma (IMCC) is the most common, accounting for approximately 78% of iCCA [60, 61]. In IMCC, FABP5 expression is significantly elevated and is positively correlated with tumor size, lymph node metastasis, vascular infiltration and TNM stage [62]. In addition, Nakagawa et al. [63] proposed that biliary tract cancer (BTC) could be categorized into two types: large BTC and small BTC. Large BTC mainly includes periportal CCA, extrahepatic CCA (eCCA) and gallbladder cancer, while small BTC includes iCCA and cholangiocellular carcinoma. Through immunohistochemical analysis of FABP5 and its related molecules in the surgical specimens of 74 patients with BTC, researchers found that the expression levels of FABP5 and PPAR-γ coactivator-1 in large BTC were greater than those in small BTC and that the prognosis of patients was worse, suggesting that there were differences in energy metabolism in CCA tissues at different anatomical locations. In summary, FABPs are closely related to the progression of CCA, and in-depth explorations of the connection between FABPs and CCA are expected to reveal new targets for CCA treatment.

### FFARs

FFARs are a group of G protein-coupled receptors (GPCRs) that can be divided into four isoforms: FFAR1-FFAR4. Free FAs (FFAs) are natural ligands for FFARs. The binding of FFAs and FFARs leads to the dissociation of the α subunit and the β-γ subunit in the intracellular coupled heterotrimeric G protein, thereby influencing intracellular signaling proteins and pathways [64]. This ultimately triggers a cascade of processes, such as metabolic disorders, inflammatory responses and immune responses. FFAR2 and FFAR3 are mainly activated by short-chain fatty acids (SCFAs), while LCFAs mainly mobilize FFAR1 and FFAR4 [65]. In recent years, an increasing number of studies have shown that FFARs participated in the progression of a variety of tumors [66, 67]. It is now well established that FFAR4 expression levels were correlated with clinical stage, tissue differentiation, and lymph node metastasis in CCA patients. Compared with that in adjacent noncancerous tissues, FFAR4 expression is notably upregulated in CCA [68]. High FFAR4 expression often implies an increase in the EMT capacity of CCA cells and a decrease in the disease-free survival and overall survival of CCA patients. Although the role of FFAR4 in the development of CCA has not been clarified, in colorectal cancer, FFAR4 can promote angiogenesis and EMT by increasing the secretion of vascular endothelial growth factor (VEGF), IL-8, and prostaglandin E2 (PGE2) [69]. Therefore, FFAR4 is expected to be a potential target for treating and improving the prognosis of CCA patients.

In conclusion, A high-fat diet accelerates the intake of exogenous lipids into fatty acids in the body. At the same time, exogenous FAs are involved in the genesis and progression of CCA through FA transporters and transport mediators, which serve as irreversible links between tumor cells and FAs. Thus, limiting high-fat diets and targeting transporters and transport mediators is anticipated to be a potential treatment approach for CCA. At present, caloric restriction (CR), which refers to the restriction of total daily caloric intake while adequately ensuring the nutrient content of an organism, has been shown to be beneficial for improving health and combating disease in a variety of animal models. Compared to ketogenic diets, CR reduces plasma lipid levels and attenuates the uptake of exogenous FAs by tumor cells [70]. It is of great clinical significance to rationally regulate the intake and transport of exogenous FAs.

## Fa Catabolic Disorders Regulate The Development Of Cca

### FAO

FAO is a process by which FAs are decomposed into various metabolites, such as CO2, H2O and a large amount of adenosine 5’-triphosphate (ATP), under aerobic conditions, with β-oxidation predominating. FAO can provide a constant source of energy for the growth and proliferation of tumor cells and drug resistance, and the content of ATP produced by FAO is twice as much as that produced by the same mass of sugars [71]. Moreover, FAO also maintains redox homeostasis and prevents lipotoxicity caused by excessive FA accumulation, creating excellent conditions for tumor progression [72–74]. FAO mainly occurs in mitochondria and involves a series of reactions. FA activation, a necessary prerequisite for FAO, is the biological process by which FAs are catalyzed in the cytoplasm by acyl-CoA synthetase to generate acyl-CoA. Acyl-CoA has a high-energy thioester bond, which not only improves the reactivity but also increases the water solubility of FAs; thus, FA activation is important for improving FA metabolic activity. Since acyl-CoA is unable to directly penetrate the inner mitochondrial membrane, acyl-CoA produced in the cytoplasm is first catalyzed by CPT-1 in the outer mitochondrial membrane to form fatty acyl-carnitine, which is then transferred to the mitochondria by means of carnitine acylcarnitine deficiency (CACT) in the inner mitochondrial membrane. Afterward, CPT-2 on the matrix side of the inner membrane reconverts acylcarnitine to acyl-CoA and carnitine. The former undergoes FAO catalyzed by fatty acid oxidase, and the latter can be transported back to the cytoplasm by CACT. FAO is closely related to the malignant phenotype of tumors, and increased FAO activity promotes the lymphatic metastasis of tumors [75]. CPT, a key enzyme of FAO, is overexpressed in diverse cancer cells and can promote cancer cell proliferation and migration. In recurrent breast cancer, FAO is enhanced and CPT1A/CPT2 expression is elevated in patients with a poor prognosis compared to those with a good prognosis. Moreover, in prostate cancer, the overexpression of CPT-1A accelerates FAO and facilitates the development and proliferation of cancer cells. A recent study revealed that inhibiting FAO with etomoxir, which acted as a potent inhibitor of CPT-1, effectively suppressed low pH-induced invasion in prostate cancer [76, 77]. Furthermore, the transcription factor PROX1 can promote lymphatic endothelial cell proliferation and lymphangiogenesis by increasing FAO activity through the upregulation of CPT1 expression [78]. Therefore, targeting FAO and its related enzymes is important for regulating malignant tumor progression. Several studies have shown that the developmental progression of CCA is closely associated with FAO. Compared with normal bile duct epithelial cells, FAO is the preferred energy source for highly proliferative CCA cells [37]. FAO and its associated proteome are enriched in the CCA cell line EGI1, and the pharmacological blockade of FAO significantly inhibits its tumorigenic capacity. The expression of acyl-CoA dehydrogenase medium chain (ACADM), an FAO-related enzyme, is increased in CCA and is correlated with the level of nuclear proliferation antigen expression. High ACADM expression could increase the proliferative capacity of CCA cells by promoting FAO. In summary, the dynamic regulation of FAO plays an important role in tumorigenesis and cancer progression, and the inhibition of FAO can be an important strategy for treating CCA. However, since relevant research is still in its infancy, elucidating the correlation between CCA and FAO and the specific underlying mechanisms still need to be further explored.

### LPO and cell death

**Fig. 4:**
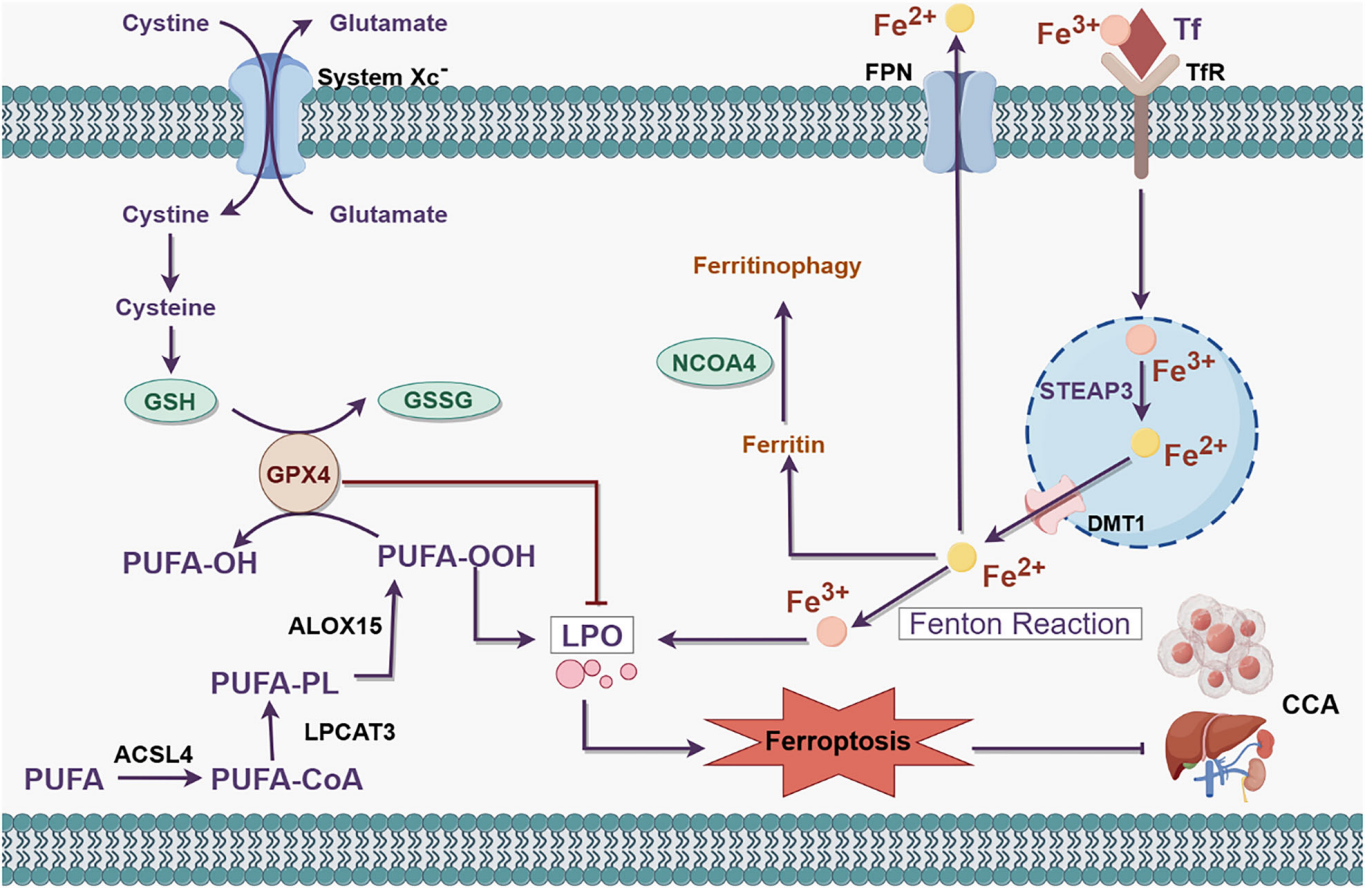
Mechanisms associated with ferroptosis in the development and progression of CCA. Ferroptosis is regulated by iron metabolism, lipid metabolism and the system Xc^-^/GPX4 pathway. System Xc^-^ regulates GSH synthesis mainly by mediating cystine uptake and glutamate output. GPX4 is able to utilize GSH to reduce PUFA-OOH to nontoxic PUFA-OH, thereby protecting cells from ferroptosis. However, during enzymatic lipid peroxidation, PUFA is anchored and elongated at the cell membrane by ACSL4 and LPCAT3, and newly produced PUFA-PL is overoxidized via toxic lipid peroxidation catalyzed by ALOX15. Additionally, TFR1 binds to Tf and transports Fe^3+^ into the cell via endocytosis; Fe^3+^ is then oxidatively reduced to Fe^2+^ by STEAP3. Fe^2+^ can transport to the cytoplasm mediated by DMT1, and then participate in the ferritin-selective autophagy process facilitated by NCOA4. At the same time, Fe^2+^ is involved in the formation of Fe^3+^ in LPO via the Fenton reaction. Ferroptosis can damage cancer cells and protect against the malignant progression of CCA. (GSH, glutathione; GSSG, glutathione disulfide; GPX4, glutathione peroxidase 4; PUFA, polyunsaturated fatty acid; ALOX15, arachidonic acid 15-lipoxygenase; ACSL4, acyl-CoA synthetase long-chain family 4; LPCAT3, lysophosphatidylcholine acyltransferase 3; PUFA-PL, polyunsaturated fatty acid-containing phospholipid; LPO, lipid peroxidation; Tf, transferrin; TfR, transferrin receptor; FPN, ferroportin; CCA, cholangiocarcinoma; STEAP3, six-transmembrane epithelial antigen of prostate 3; DMT1, divalent metal transporter 1; NCOA4, nuclear receptor coactivator 4).

LPO is the process by which lipids are oxidized to produce lipid peroxides and consists of two types: enzymatic LPO and nonenzymatic LPO. LPO has been shown to be involved in a variety of cell death processes, such as apoptosis, autophagy and iron death, and is closely associated with tumors, inflammation and neurodegenerative diseases. Enzymatic LPO is mediated by the lipoxygenase (LOX), cyclooxygenase (COX), and CYP450 families, which catalyze the deoxygenation of free and esterified PUFAs to generate various lipid hydroperoxides; nonenzymatic lipid peroxidation, on the other hand, is usually defined as a chain reaction driven by lipid free radicals [79]. There is a close link between LPO and oxidative stress. Oxidative stress is a state of imbalance between the oxidative and antioxidant systems in the body. Compared to normal cells, cancer cells usually exhibit abnormal redox homeostasis. Specifically, the excessive proliferation of cancer cells is often accompanied by the abnormal production of reactive oxygen species (ROS), and a certain level of ROS can promote tumor progression. However, excessive ROS are cytotoxic. To adapt to high levels of ROS and maintain redox homeostasis, cancer cells tend to increase the activity of antioxidant transcription factors in the body and ultimately maintain ROS below this threshold to avoid oxidative stress-induced cell death [80, 81]. Hepatic schistosomiasis is a class I carcinogen of CCA. Chronic inflammation induced by liver fluke infection leads to cellular damage and homeostatic dysregulation through the production of large amounts of oxidizing free radicals capable of destroying biomolecules, leading to LPO [82]. In addition, patients with opisthorchiasis viverrini exhibit high urinary excretion of LPO-derived vinyl DNA adducts (εdA and εdC), suggesting a high degree of DNA damage in the hepatic biliary tract, which is closely related to inflammation-induced oxidative stress and the accumulation of LPO-derived DNA damage [83]. In an environment of high levels of oxidative damage, bile duct epithelial cells can undergo malignant transformation to CCA. Therefore, maintaining redox homeostasis is essential for CCA. In recent years, an increasing number of studies have shown that ferroptosis has great potential in the treatment of drug-resistant tumors and is expected to become a novel therapeutic modality (Fig.4). Ferroptosis refers to the process by which Fe^2+^-catalyzed cell death occurs when lipid ROS overaccumulate and is an iron-dependent nonapoptotic cell death characterized by elevated lipid peroxide and decreased glutathione peroxidase levels [84]. LPO-induced ferroptosis plays a crucial role in the occurrence and development of CCA [85]. Ferroportin and transferrin levels have been reported to be lower in CCA tissues than in adjacent normal tissues, while the transferrin receptor level was higher, and the prognosis of CCA patients has been shown to deteriorate with increased iron deposition [86]. Furthermore, JUND/lincoo976 was found to inhibit ferroptosis in CCA cells by regulating the miR-3202/GPX4 axis [87]. Conversely, the downregulation of the expression of SLC7A11, GPX4, SOD-1 and SOD-2 in CCA cells can induce ferroptosis and delay tumor progression [88]. Thus, LPO and ferroptosis are closely related to the development of CCA. PUFAs, as the preferred substrates of LPO, especially arachidonic acid (AA) and adrenic acid, are highly likely to disrupt the lipid bilayer structure and affect membrane function [89]. Long-chain acyl-CoA synthetase 4 (ACSL4) is a key enzyme in PUFA activation and is also considered a key protein in ferroptosis. By regulating FA metabolism, ACSL4 not only participates in a wide range of biological processes, such as energy metabolism, endoplasmic reticulum stress and TME formation but also regulates the accumulation of LPO substrates to influence iron death [90]. Currently, ACSL4 has become a biomarker for the diagnosis and treatment of a variety of tumors. Depending on the tumor type and tissue environment, ACSL4 can exert either pro- or antitumorigenic effects. In lung adenocarcinoma and breast cancer, ACSL4 is considered a tumor suppressor, while in HCC and colon cancer, ACSL4 promotes tumor progression [91–94]. In response to the relationship between CCA and ACSL4, Liu S et al. [95] reported for the first time that ACSL4 is closely related to ferroptosis in CCA. ACSL4 is highly expressed in CCA and correlated with poor prognosis. When ACSL4 is knocked down in CCA cells, ROS accumulation is reduced, ferroptosis is inhibited, and the proliferation and invasion of CCA cells are enhanced. The addition of erastin (a ferroptosis activator) reverses ROS accumulation, accelerates the process of iron death, and attenuates the malignant progression of CCA. At the same time, quercetin, a natural flavonoid, can delay the malignant progression of iCCA by inducing ferroptosis and invasion via the NF-κB pathway and has great potential for the prevention and treatment of CCA [96]. Therefore, increasing the oncogenic effect of ferroptosis for tumor prevention and treatment is highly important, and in-depth studies of the relationship between LPO and CCA is expected to reveal a new target for tumor therapy.

## Differences In Fa Categories And Composition Ratio Have Multiple Effects On The Onset And Advancement Of Cca

Based on the number of carbon‒carbon double bonds, FAs are classified into saturated fatty acids(SFAs), monounsaturated fatty acids (MUFAs) containing one double bond, and PUFAs containing at least two double bonds. PUFAs are further classified into various categories on the basis of the number of carbons counted from the methyl end of the first double bond, commonly known as ω-3 PUFAs and ω-6 PUFAs. Since changes in the type and content of intracellular FAs affect the structure and function of cell membranes, the ability of cells to take up glucose, and the level of intracellular ATP, in-depth investigations of the multiple effects of differences in FA types and composition ratios on cancer cells are highly important for the prevention and treatment of tumors. Compared with those of normal cells, the cell membrane of cancer cells is characterized by an increased ratio of MUFA/SFA acyl side chains and MUFA/PUFA acyl side chains in terms of the FA composition ratio, which plays an essential role in decreasing lipotoxicity and sensitivity to iron death [73]. Currently, an increasing number of studies have confirmed that differences in FA types and composition ratios have multiple effects on the occurrence and development of CCA(Table 2).

**Table. 2:** Multiple effects of FA types and composition ratios on CCA.

Type of FAs	Representative	Research	Ref.
SFAs	Butyric acid	Butyrate induces cilia formation and growth inhibition in CCA cells, and the combination of butyrate with HDAC6 inhibitors halts cell growth, migration, and EMT in CCA at lower doses.	[99]
	Palmitic acid	Palmitic acid has a pro-oncogenic effect on CCA, and supplementation with palmitic acid in CCA cells with ACC1 knockdown restores the invasive migration ability of the cells.	[15]
MUFAs	Oleic acid	Oleic acid activates FA uptake and FAO through the oleate-PPARγ-FABP4 positive feedback loop to promote lymph node colonization by CCA cells.	[106]
ω-3 PUFAs	Alpha-linolenic acid, eicosapentaenoic acid, docosahexaenoic acid	ω-3 PUFAs inhibit proliferation and metastasis of CCA cells by inhibiting the expression of twist.	[102]
		ω-3 PUFAs facilitate rapid dephosphorylation of GSK-3β, leading to β-catenin degradation in CCA cells, and inhibit 15-PGDH expression induced by COX-2 to delay malignant progression of CCA.	[110]
		ω-3 PUFAs reduced miR26a/b expression through the negative regulation of the transcription factor c-myc, targeted enhancement of 15-PGDH activity, and downregulation of PGE2 expression, ultimately halting the progression of CCA.	[113]
ω-6 PUFAs	Arachidonic acid	Arachidonic acid is synthesized into LT by 5-LOX, and LT is further converted into LX in a process catalyzed by 15-LOX. The prognosis of CCA patients is negatively correlated with the expression of 5-LOX and positively correlated with the expression of 15-LOX.	[115]
		Arachidonic acid generates PG under the catalysis of COX-2, and PG contributes to accelerating the progression of CCA.	[117]
	Linoleic acid	Linoleic acid disorders lead to the transformation of intrahepatic bile duct stones into iCCA.	[124]
		Linoleic acid increases the metabolic adaptation and antitumor immune effects of CD8 + T cells and increase immune efficacy as an enhancer of adoptive cell transfer therapy.	[126]
ω-6/ω-3 PUFAs		The recommended dietary ratio for Chinese residents is 4–6:1, while the ratio is much higher in the Western diet due to a lack of foods rich in ω-3 PUFAs.	[128]
		A high dietary ω-6/ω-3 PUFA ratio is associated with an increased risk of gallstone incidence.	[133]

### SFAs

SFAs are FAs without unsaturated double bonds in the carbon chain and are mainly derived from milk, dietary fiber, vegetable oils and peanut oil. SFAs have a variety of beneficial biological functions; for instance, they can exert oncostatic effects by blocking the cell cycle, inducing apoptosis, modulating cell growth-related signaling pathways and affecting the expression of related genes and the phosphorylation levels of transcription factors [97]. Butyric acid, which is produced by the fermentation of intestinal fiber bacteria as a saturated short-chain FA, is also known as a potential carcinogenic FA. In the CCA, butyric acid usually exerts a tumor suppressor effect in the form of butyrate and is closely related to cellular cilia. Cellular cilia, as “signaling antennae” protruding from the cell surface, are responsible for sensing and transmitting various extracellular signaling stimuli and regulating cellular life activities. Normal bile duct epithelial cells can deliver bile stimuli to cells via primary cilia and participate in the regulation of secretion, cell proliferation and apoptosis signaling pathways. Mansini AP et al. [98] found that the primary cilia of CCA cells were dysfunctional and that the biliary cilia of CCA cells were significantly reduced compared to those of normal cells. The absence of cilia in the biliary epithelium can lead to the excessive proliferation of CCA cells, changes in fluid secretion and absorption, and ultimately a malignant phenotype. Therefore, cilia can serve as a type of tumor suppressor organelle that regulates tumor development and progression. Restoring primary cilia is a potential therapeutic approach for treating CCA. Pant K et al. [99] conducted a comprehensive study and found that compared with control cells, CCA cells treated with butyrate were more capable of forming cilia, had increased expression of acetylated microtubule proteins, and showed reduced proliferation and migration ability. Histone deacetylase 6 (HDAC6) is an enzyme that regulates epigenetic modifications and can induce CCA development by promoting cilia loss, making it an independent prognostic factor for CCA [100]. Butyrate can enhance the inhibitory effects of HDAC6 inhibitors on CCA cell proliferation and migration. Therefore, regulating the in situ cilia and histone acetylation of CCA cells by butyrate is highly important for the prevention and treatment of CCA. However, not all SFAs can inhibit tumor progression. Pascual G et al. [101] investigated the mechanism of FA in relation to tumor metastasis by in situ transplantation of FA-treated human oral squamous carcinoma cells into mice and found that tumor cells treated with PA could maintain long-term metastatic ability even after the FA supply was interrupted, which was related to the mechanism by which PA could promote tumor cell metastasis through Schwann cells and could form a neural network around the tumor to facilitate further tumor cell dissemination by altering epigenetics. PA has a pro-oncogenic effect on CCA, and supplementation with PA and malonyl-CoA in CCA cells with ACC1 knockdown restores the invasive migration ability of the cells [15]. Although there has been little research on the relationship between SFAs and CCA thus far, the different effects of butyric acid and PA on CCA suggest that the proportion of SFAs that regulate the procancer and anticancer effects in CCA cells is of great importance for cancer prevention.

### UFAs

#### MUFAs

Unsaturated fatty acids (UFAs) are typically generated from the desaturation of SFAs. Stearoyl-CoA desaturase-1 (SCD-1) is the key enzyme in the synthesis of MUFAs and can convert SFAs, particularly palmitoyl-CoA or stearoyl-CoA, into MUFAs, such as palmitoleic acid or oleic acid. SCD-1 has been found to be highly expressed in various tumors, including gastric cancer, pancreatic cancerand lung cancer [13, 102, 103]. In CCA, SCD-1 expression is upregulated, and the highly specific SCD-1 inhibitor SSI-4 can retard CCA growth and proliferation and has synergistic antitumor effects with GEM and cisplatin [104]. As a typical MUFA, oleic acid plays a significant role in promoting tumor initiation and progression. For instance, in ovarian cancer, oleic acid not only activates PPARα to induce cell cycle progression but also accelerates glycolysis through the BRD4-L-MYC-GLUT axis to promote ovarian cancer cell proliferation and survival [105]. Due to their strong metastatic potential, CCA cells can spread to distant sites through direct invasion, lymph node metastasis, and hematogenous dissemination. Therefore, for CCA patients with lymph node metastasis, postoperative complete lymph node dissection is particularly crucial for prognosis. I t has been reported that oleic acid could activate FA uptake and FAO through the oleate-PPARγ-FABP4 positive feedback loop to promote lymph node colonization by CCA cells [106]. Currently, CD36 monoclonal antibodies have been shown to significantly inhibit tumor metastasis without side effects in oral squamous cell carcinoma models and are expected to be useful for treating CCA [41]. Thrombospondin-1 (THBS-1) can predict GEM chemosensitivity in iCCA patients and can be used as a chemotherapeutic sensitizer to improve drug resistance [107]. In mechanistic terms, this effect may be related to the influence of THBS-1 on oleic acid uptake via CD36. Thus, regulating MUFA levels, especially oleic acid content, is highly important to ameliorate the malignant progression of CCA.

#### ω-3 PUFAs

ω-3 PUFAs are PUFAs with the first unsaturated bond at the 3rd position of the methyl end of the carbon chain and mainly include alpha-linolenic acid (ALA), eicosapentaenoic acid (EPA) and docosahexaenoic acid (DHA). ω-3 PUFAs are commonly found in fatty deep-sea cold fish and flaxseed oil. Epidemiological investigations have shown that ω-3 PUFAs reduced the incidence of malignant tumors in humans and that increasing the intake of ω-3 PUFAs in the diet could inhibit the development of a variety of tumors, alleviate the symptoms of cachexia in patients with malignant tumors, reduce body weight loss or even increase body weight [108]. Moreover, ω-3 PUFAs are used as nontoxic adjuvant therapeutic agents for the treatment of tumors. High levels of ω-3 PUFAs exert a significant inhibitory effect on CCA. Lin et al. [109] established a zebrafish model of ICC and discovered that fish oil rich in ω-3 PUFAs not only suppressed the migration and invasion ability of CCA cells by downregulating the expression of MMP7, MMP9, TWIST, Snail-1, and VEGFA but also disrupted the G2/M phase of the cell cycle and suppressed the proliferation of CCA cells. The inhibitory effect of ω-3 PUFAs on CCA may be associated with the Wnt/β-catenin and COX-2 signaling pathways. The oncogenic effects of DHA and EPA on CCA were found to be dependent on both dose and time. They not only facilitate rapid dephosphorylation of GSK-3β, leading to β-catenin degradation in CCA cells but also inhibit 15-hydroxyprostaglandin dehydrogenase (15-PGDH) expression induced by COX-2, thus delaying malignant progression [110]. When combined with the COX-2 selective inhibitor NS-398, DHA significantly decreases β-catenin and COX-2 expression levels in CCA cells while markedly inhibiting cell growth [111, 112]. Additionally, Yao L et al. [113] reported that ω-3 PUFAs reduced miR26a/b expression through the negative regulation of the transcription factor c-myc, targeted enhancement of 15-PGDH activity, and downregulation of PGE2 expression, ultimately halting the progression of CCA. Resolvin D1 is an endogenous lipid mediator synthesized from ω-3 PUFAs by an enzymatic process involving 15-LOX and 5-LOX. The resolvin D1 concentration is negatively correlated with tumor stage. Compared with that in patients with benign biliary diseases, the serum level of Resolvin D1 is significantly lower in patients with CCA [114]. The serum Resolvin D1 concentration is helpful for the early identification of benign biliary diseases and CCA. Therefore, regulating ω-3 PUFA metabolites as well as their signaling pathways holds great promise for preventing CCA. Therefore, regulating the ω-3 PUFA content, metabolites and related signaling pathways is highly important for the prevention and control of CCA.

#### ω-6 PUFAs

Mainly include linoleic acid (LA) and AA, often increase the risk of malignant tumorigenesis, in contrast to the anti-oncogenic effects of ω-3 PUFAs. Several studies have shown that ω-6 PUFAs participated in the progression of CCA through multiple mechanisms. AA is one of the most abundant and widely distributed PUFAs in the body. It can produce metabolites such as PG, thromboxane (TX), lipoxin (LX) and leukotriene (LT) through three metabolic enzyme families: COX, LOX and CYP450. AA can be synthesized into LT by 5-LOX, and LT can be further converted into LX in a process catalyzed by 15-LOX. LT and LX have opposite biological functions: LT promotes inflammation and tumor progression, whereas LX, as a lipid-resolving mediator, attenuates inflammation and slows tumor progression; thus, it is important to maintain their balance during disease progression. The prognosis of CCA patients is negatively correlated with the expression of 5-LOX and positively correlated with the expression of 15-LOX, and when both are coexpressed, patients have a favorable prognosis [115]. Chiruton, an inhibitor of 5-LOX, reverses EMT in CCA cells by decreasing LT accumulation and increasing LX production. Therefore, targeting 5-LOX and 15-LOX to regulate lipid metabolism could be a potential modality for treating CCA. Moreover, COX-2 is highly expressed in the bile duct tissues of CCA patients and precancerous lesions. When COX-2 is inhibited, CCA cells exhibit decreased proliferation and increased apoptosis [116]. AA can generate PG under the catalysis of COX-2, and PG contributes to accelerating the progression of CCA. Wu T et al. [117] reported that the incubation of CCA cells with hepatocyte growth factor (HGF), IL-6 and PGE2 resulted in the rapid release of AA and the production of PG from CCA cells, significantly increasing the proliferation of CCA cells. Recent studies have demonstrated that PG was involved in the progression of CCA through multiple mechanisms. For example, PGE induces CCA development by upregulating β-catenin, c-myc and snail expression through the EP3-4R/Src/EGFR/PI3K/AKT/GSK-3β pathway [118]. In addition, PGE2 can facilitate the phosphorylation of extracellular signal-regulated kinases and cAMP response element-binding proteins through the activation of the prostaglandin E receptor 1 (PTGER1) signaling pathway, upregulating MMP-2 expression and inducing EMT in CCA cells [119]. SC-51322, a selective antagonist of PTGER1, can delay the malignant progression of CCA cells by inhibiting PGE2. The COX-2 inhibitors meloxicam and xanthohumol can be used as alternative treatments for CCA, delaying the occurrence and progression of CCA by downregulating PGE2 expression [120]. The expression of 15-PGDH, a key enzyme involved in the catabolism of PGE2, is closely related to the malignant process of CCA. It was found that miR-21 could enhance PGE2 accumulation by inhibiting 15-PGDH and that increased PGE2 signaling could further stimulate miR-21 transcription, ultimately forming a vicious cycle leading to the accumulation of PGE2 in vivo and accelerating the development of CCA [121]. Thus, targeting the enzymes and signaling pathways related to AA metabolic processes is highly important for the treatment of CCA.

LA has multiple effects on different types of tumor cells [122, 123]. Regarding the relationship between LA and CCA, it has been shown that LA disorders could lead to the transformation of intrahepatic bile duct stones into iCCA [124]. Bile duct stones are one of the risk factors for iCCA. Stones located in the lower end of the common bile duct are usually referred to as extrahepatic bile duct stones, while stones distributed in the bile ducts within the liver lobes are referred to as intrahepatic bile duct stones. The level of LA in intrahepatic bile duct stones is almost twice as high as that in iCCA. The decrease in LA might be associated with the upregulation of peripheral blood γ-glutamyl transpeptidase and alkaline phosphatase expression, as well as with the downregulation of the expression of other hepatic functional proteins, such as proalbumin, blood urea nitrogen, and creatinine. These findings suggest that the long-term monitoring of FA metabolism and hepatic function protein indices in patients with intrahepatic bile duct stones provides important guidance for predicting cancerous changes in intrahepatic bile duct stones. It has been reported that LA could exert cancer-suppressive effects with the help of adoptive cell transfer therapy (ACT), which was a new type of immune cell therapy in which the patient’s own immune cells were collected, cultured and modified in vitro, the target killing function was increased, and the agent was subsequently infused into the patient’s body to eliminate tumor cells [125]. Poor metabolic status is a major obstacle to the low efficacy of ACT, and certain specific lipids can damage the integrity of CD8^+^ T cell mitochondria, leading to defective antitumor immune responses. LA can increase the metabolic adaptation and antitumor immune effects of CD8^+^ T cells and increase immune efficacy as an enhancer of ACT [126]. In addition, conjugated LA, as a mixture of isomers of LA, has biological functions such as promoting apoptosis and inhibiting tumor cell proliferation [127]. Most conjugated LA can exert antitumor activity through the regulation of PPAR. The PPAR pathway is closely related to CCA. Although there are no relevant reports of conjugated LA in CCA, the anticancer effects of conjugated LA are highly relevant to CCA. An in-depth understanding of the causal relationship between ω-6 PUFA metabolism disorders and the development and progression of CCA may provide important therapeutic targets for CCA.

#### ω-6/ω-3 PUFAs

The balance of ω-6 and ω-3 PUFA contents is crucial for maintaining normal metabolism, as they often compete in metabolic pathways. At present, the optimal recommended ω-6/ω-3 PUFA ratio has not been standardized internationally. The recommended dietary ratio for Chinese residents is 4–6:1. However, the typical Western diet lacks foods rich in ω-3 PUFAs, resulting in a much higher ω-6/ω-3 PUFA ratio than the recommended value [128]. An increased ω-6/ω-3 PUFA ratio leads to various diseases, such as autoimmune diseases, cancers, inflammation and psychological disorders [129–131]. Dierge E et al. [132] reported that a diet rich in ω-3 PUFAs could significantly slow tumor growth in mice, while increased dietary PUFA intake could promote the occurrence of LPO and ferroptosis in cancer cells. Thus, rational regulation of the amount and type of FAs in the diet is highly important for disease prevention and treatment. Cholelithiasis is an important risk factor for CCA, with a global prevalence of approximately 10%, of which the prevalence in Europe and the United States is much greater than that in Asia (up to more than 20%). A typical Western diet has been shown to increase the risk of gallstone disease. Campos-Perez W et al. [133] conducted a cross-sectional study of dietary intake in 54 women with gallstone disease and 75 women without gallstone disease from the state of Mexico and found that a high dietary ω-6/ω-3 PUFA ratio and an excessive intake of simple carbohydrates are associated with an increased risk of gallstone incidence. Therefore, the rational regulation of the dietary ω-6/ω-3 PUFA ratio may have a positive role in reducing biliary tract disease and its deterioration.

## Perspectives

The reprogramming of FA metabolism plays an important role in the development and progression of CCA. Compared with normal bile duct epithelial cells, CCA cells meet the need for tumor cell deterioration by enhancing FA metabolism. FAs are maintained mainly through two pathways: exogenous intake and endogenous synthesis. The vast majority of normal cells preferentially use exogenous FAs; in other words, FA de novo synthesis is often inhibited by dietary lipids in normal cells. However, for cancer cells, FA de novo synthesis is the main way for organisms to obtain FAs. The expression levels of ACC and FASN, key enzymes of FA de novo synthesis pathway, are closely related to the development and progression of CCA, and it has been reported that a variety of drugs could affect the FA synthesis and metabolism to exert oncogenic effects by regulating ACC and FASN. Moreover, Acetyl-CoA, an important raw material in FA de novo synthesis, is closely related to the expression of ACLY and ACSS. A reduction in acetyl-CoA production is important for the inhibition of FA anabolism. ACLY, an important bridge between glucose metabolism and lipid metabolism, is highly expressed in a variety of tumors. ACLY is involved in various oncogenic signaling cascade responses. A recent study revealed that the intraperitoneal injection of the ACLY inhibitor SB-204990 in mice attenuated tumorigenicity [134].Berberine inhibits ACLY-induced lipid metabolism disorders and delays the malignant process of pancreatic cancer [135]. Therefore, ACLY inhibitors are highly important for tumor control. As the current ACLY inhibitors still have limitations, such as poor physicochemical properties and a lack of selectivity and cytotoxicity, they are mostly applied to dyslipidemia-related diseases. An in-depth study of the pathogenesis of ACLY in CCA and the design of safer and more effective inhibitors are necessary but challenging for the prevention and treatment of CCA [136]. In addition, sterol regulatory element binding proteins (SREBPs) are important transcription factors involved in lipid metabolism. They consist of three isoforms: SREBP1a, SREBP1c, and SREBP2. SREBP1 is mainly involved in FA synthesis, while SREBP2 is responsible for cholesterol metabolism. SREBPs are often associated with the PI3K/AKT/mTOR signaling pathway and are involved in the development of various tumors, and the inhibition of SREBPs and their downstream genes can significantly delay the malignant progression of tumors [137, 138]. Cordycepin is a nucleoside antibiotic isolated from fungi. In CCA, cordycepin can inhibit SREBP1-mediated FA synthesis and exert oncogenic effects by suppressing the AKT/mTOR signaling pathway through the inhibition of ERO1A [139]. The development of new drugs for the treatment of CCA and improvements in prognosis with the help of transcription factors related to the regulation of FA metabolism has a promising future in disease prevention and treatment. However, FASN-dependent endogenous synthesis of FAs is not a common feature of all liver tumors, the development of iCCA is not sensitive to a lack of FASN, and tumor growth is dependent mainly on exogenous FA uptake, suggesting that inhibition of exogenous FA uptake may be a new therapy for iCCA. Modulating FA acquisition pathways according to the metabolic characteristics of CCA may provide new ideas for the diagnosis and treatment of CCA.

FAs enter cancer cells to exert various effects with the assistance of transporter carriers. ω-3 PUFAs mainly exert anti-oncogenic effects, while ω-6 PUFAs mainly have pro-oncogenic effects, and SFAs have different mechanisms of action on CCA according to their class. FAs can synthesize other lipids to participate in tumor metabolic reprogramming. Therefore, regulating FA transport and maintaining a balanced proportion of various FAs in the body are essential factors associated with the occurrence and development of CCA. In addition, the rational regulation of the content and type of FAs in the diet is a selective and complementary antitumor approach. For patients with malignant tumors, increased intake of foods rich in ω-3 PUFAs, such as deep-sea cold fish, walnuts and flaxseed oil, is recommended. FAs can also supply energy for CCA cell growth through catabolic oxidation. Abnormal lipid catabolism, such as LPO and ferroptosis, is closely associated with the malignant progression, drug resistance and poor prognosis of CCA. In recent years, targeting ferroptosis has gradually become a new therapy. Ferroptosis inducers not only increase the cytotoxicity of chemotherapeutic agents and attenuate drug resistance but also effectively synergize with radiotherapy to increase sensitivity and improve patient prognosis [140, 141]. Therefore, regulating ferroptosis is highly important for tumor prevention and treatment.

In summary, FA metabolic reprogramming is highly important for the development and progression of CCA. Targeting FA metabolism has gradually become an important method for tumor treatment and improving drug resistance. At present, it has been effective in targeting fatty acid metabolism(Table 3). However, since therapeutic modalities targeting some metabolic targets have not yet been effectively developed and clinically applied, in-depth investigations of the relationship between FA metabolic reprogramming and the development of CCA, as well as the discovery of more therapeutically valuable potential targets, have become urgently needed. Overall, with further research, targeted FA metabolism therapy has broad application prospects for suppressing the development and progression of CCA.

**Table. 3:** Targeted FA metabolism to inhibit the occurrence and progression of CCA.

Clinical treatment	Research	Ref.
TOFA	TOFA impairs the endogenous synthesis capacity of FAs by down-regulating ACC expression and inhibits the proliferation of CCA cells in a dose-dependent manner.	[19]
TVB-2640	TVB-2640 has entered the phase 2 clinical trial stage and has been confirmed to have safe and effective cancer inhibitory effects.	[32]
Caloric restriction	Caloric restriction reduces plasma lipid levels and attenuates the uptake of exogenous FAs by tumor cells.	[70]
Quercetin	Quercetin can delay the malignant progression of iCCA by inducing ferroptosis and invasion via the NF-κB pathway.	[96]
Thrombospondin-1	Thrombospondin-1 which affects cellular uptake of oleic acid via CD36, can be used to predict GEM chemosensitivity in patients with iCCA and can be used as a chemotherapy sensitiser to improve drug resistance.	[107]
NS-398	When combined with the COX-2 selective inhibitor NS-398, DHA significantly decreases β-catenin and COX-2 expression levels in CCA cells while markedly inhibiting cell growth.	[111]
Meloxicam and xanthohumol	The COX-2 inhibitors meloxicam and xanthohumol delay the onset and progression of CCA by down-regulating the expression of PGE2, and may be used as alternative therapies for the treatment of CCA.	[120]
SC-51322	SC-51322, a selective antagonist of PTGER1, can delay the malignant progression of CCA cells by inhibiting PGE2.	[119]
Cordycepin	Cordycepin can inhibit SREBP1-mediated FA synthesis and exert oncogenic effects by suppressing the AKT/mTOR signaling pathway through the inhibition of ERO1A in CCA.	[139]

## Data Availability

Not applicable.

## References

[1] Valle JW, Kelley RK, Nervi B, Oh DY, Zhu AX. Biliary tract cancer. Lancet Lond Engl 2021;397:428–44. 10.1016/S0140-6736(21)00153-7 33516341

[2] Rizvi S, Khan SA, Hallemeier CL, Kelley RK, Gores GJ. Cholangiocarcinoma - evolving concepts and therapeutic strategies. Nat Rev Clin Oncol 2018;15:95–111. 28994423 10.1038/nrclinonc.2017.157 PMC5819599

[3] Høgdall D, Lewinska M, Andersen JB. Desmoplastic Tumor Microenvironment and Immunotherapy in Cholangiocarcinoma. Trends Cancer. 2018;4:239–55. 29506673 10.1016/j.trecan.2018.01.007

[4] Cillo U, Fondevila C, Donadon M, Gringeri E, Mocchegiani F, Schlitt HJ, et al. Surgery for cholangiocarcinoma. Liver Int J Int Assoc Study Liver. 2019;39:143–55. 10.1111/liv.14089 PMC6563077 30843343

[5] Sun Z, Han X, You W, Tang J, Xu J, Ye B, et al. Adjuvant therapy for cholangiocarcinoma after surgery and prognosis factors for cholangiocarcinoma: A single-center retrospective cohort study. Front Oncol. 2023;13:1116338. 37007129 10.3389/fonc.2023.1116338 PMC10063974

[6] Kelley RK, Bridgewater J, Gores GJ, Zhu AX. Systemic therapies for intrahepatic cholangiocarcinoma. J Hepatol 2020;72:353–63. 31954497 10.1016/j.jhep.2019.10.009

[7] Sarcognato S, Sacchi D, Fassan M, Fabris L, Cadamuro M, Zanus G, et al. Cholangiocarcinoma. Pathol - J Ital Soc Anat Pathol Diagn Cytopathol 2021;113:158–69. 10.32074/1591-951X-252 PMC8299326 34294934

[8] Hanahan D, Weinberg RA. Hallmarks of cancer: the next generation. Cell. 2011;144:646–74. 21376230 10.1016/j.cell.2011.02.013

[9] Snaebjornsson MT, Janaki-Raman S, Schulze A. Greasing the Wheels of the Cancer Machine: The Role of Lipid Metabolism in Cancer. Cell Metab 2020;31:62–76. 31813823 10.1016/j.cmet.2019.11.010

[10] Rysman E, Brusselmans K, Scheys K, Timmermans L, Derua R, Munck S, et al. De novo lipogenesis protects cancer cells from free radicals and chemotherapeutics by promoting membrane lipid saturation. Cancer Res. 2010;70:8117–26. 20876798 10.1158/0008-5472.CAN-09-3871

[11] Hunkeler M, Hagmann A, Stuttfeld E, Chami M, Guri Y, Stahlberg H, et al. Structural basis for regulation of human acetyl-CoA carboxylase. Nature. 2018;558:470–4. 29899443 10.1038/s41586-018-0201-4

[12] Svensson RU, Parker SJ, Eichner LJ, Kolar MJ, Wallace M, Brun SN, et al. Inhibition of acetyl-CoA carboxylase suppresses fatty acid synthesis and tumor growth of non-small-cell lung cancer in preclinical models. Nat Med. 2016;22:1108–19. 27643638 10.1038/nm.4181 PMC5053891

[13] Li C, Zhang L, Qiu Z, Deng W, Wang W. Key Molecules of Fatty Acid Metabolism in Gastric Cancer. Biomolecules. 2022;12:706. 35625633 10.3390/biom12050706 PMC9138239

[14] Lally JSV, Ghoshal S, DePeralta DK, Moaven O, Wei L, Masia R, et al. Inhibition of Acetyl-CoA Carboxylase by Phosphorylation or the Inhibitor ND-654 Suppresses Lipogenesis and Hepatocellular Carcinoma. Cell Metab. 2019;29:174–.e5. 30244972 10.1016/j.cmet.2018.08.020 PMC6643297

[15] Saisomboon S, Kariya R, Boonnate P, Sawanyawisuth K, Cha’on U, Luvira V, et al. Diminishing acetyl-CoA carboxylase 1 attenuates CCA migration via AMPK-NF-κB-snail axis. Biochim Biophys Acta Mol Basis Dis 2023;1869:166694. 36972768 10.1016/j.bbadis.2023.166694

[16] Grahame Hardie D. AMP-activated protein kinase: a key regulator of energy balance with many roles in human disease. J Intern Med. 2014;276:543–59. 24824502 10.1111/joim.12268 PMC5705060

[17] Hardie DG, Pan DA. Regulation of fatty acid synthesis and oxidation by the AMP-activated protein kinase. Biochem Soc Trans 2002;30:1064–70. 12440973 10.1042/bst0301064

[18] Gu S, Song X, Xie R, Ouyang C, Xie L, Li Q, et al. Berberine inhibits cancer cells growth by suppressing fatty acid synthesis and biogenesis of extracellular vesicles. Life Sci. 2020;257:118122. 32702446 10.1016/j.lfs.2020.118122

[19] Boonnate P, Kariya R, Saranaruk P, Cha’on U, Sawanyawisuth K, Jitrapakdee S, et al. Five-(Tetradecyloxy)-2-furoic Acid Alleviates Cholangiocarcinoma Growth by Inhibition of Cell-cycle Progression and Induction of Apoptosis. Anticancer Res. 2021;41:3389–400. 34230134 10.21873/anticanres.15126

[20] Bruning U, Morales-Rodriguez F, Kalucka J, Goveia J, Taverna F, Queiroz KCS, et al. Impairment of Angiogenesis by Fatty Acid Synthase Inhibition Involves mTOR Malonylation. Cell Metab. 2018;28:866–.e15. 30146486 10.1016/j.cmet.2018.07.019 PMC8057116

[21] Galbraith L, Leung HY, Ahmad I. Lipid pathway deregulation in advanced prostate cancer. Pharm Res. 2018;131:177–84. 10.1016/j.phrs.2018.02.022 29466694

[22] Jiang L, Wang H, Li J, Fang X, Pan H, Yuan X, et al. Up-regulated FASN expression promotes transcoelomic metastasis of ovarian cancer cell through epithelial-mesenchymal transition. Int J Mol Sci. 2014;15:11539–54. 24979135 10.3390/ijms150711539 PMC4139798

[23] Tomacha J, Dokduang H, Padthaisong S, Namwat N, Klanrit P, Phetcharaburanin J, et al. Targeting Fatty Acid Synthase Modulates Metabolic Pathways and Inhibits Cholangiocarcinoma Cell Progression. Front Pharm. 2021;12:696961. 10.3389/fphar.2021.696961 PMC8371458 34421595

[24] Su WJ, Lu PZ, Wu Y, Kalpana K, Yang CK, Lu GD. Identification of Key Genes in Purine Metabolism as Prognostic Biomarker for Hepatocellular Carcinoma. Front Oncol. 2020;10:583053. 33520699 10.3389/fonc.2020.583053 PMC7841304

[25] Kristensen LS, Andersen MS, Stagsted LVW, Ebbesen KK, Hansen TB, Kjems J. The biogenesis, biology and characterization of circular RNAs. Nat Rev Genet. 2019;20:675–91. 31395983 10.1038/s41576-019-0158-7

[26] Yu X, Tong H, Chen J, Tang C, Wang S, Si Y, et al. CircRNA MBOAT2 promotes intrahepatic cholangiocarcinoma progression and lipid metabolism reprogramming by stabilizing PTBP1 to facilitate FASN mRNA cytoplasmic export. Cell Death Dis. 2023;14:20. 36635270 10.1038/s41419-022-05540-y PMC9837196

[27] Mavangira V, Sordillo LM. Role of lipid mediators in the regulation of oxidative stress and inflammatory responses in dairy cattle. Res Vet Sci. 2018;116:4–14. 28807478 10.1016/j.rvsc.2017.08.002

[28] Zhang B, Zhou BH, Xiao M, Li H, Guo L, Wang MX, et al. KDM5C Represses FASN-Mediated Lipid Metabolism to Exert Tumor Suppressor Activity in Intrahepatic Cholangiocarcinoma. Front Oncol. 2020;10:1025. 32714863 10.3389/fonc.2020.01025 PMC7344276

[29] Lemster AL, Sievers E, Pasternack H, Lazar-Karsten P, Klümper N, Sailer V, et al. Histone Demethylase KDM5C Drives Prostate Cancer Progression by Promoting EMT. Cancers. 2022;14:1894. 35454801 10.3390/cancers14081894 PMC9032772

[30] Shen HF, Zhang WJ, Huang Y, He YH, Hu GS, Wang L, et al. The Dual Function of KDM5C in Both Gene Transcriptional Activation and Repression Promotes Breast Cancer Cell Growth and Tumorigenesis. Adv Sci Weinh Baden -Wurtt Ger. 2021;8:2004635. 10.1002/advs.202004635 PMC8097366 33977073

[31] Chen Y, Xu X, Wang Y, Zhang Y, Zhou T, Jiang W, et al. Hypoxia-induced SKA3 promoted cholangiocarcinoma progression and chemoresistance by enhancing fatty acid synthesis via the regulation of PAR-dependent HIF-1a deubiquitylation. J Exp Clin Cancer Res CR. 2023;42:265. 37821935 10.1186/s13046-023-02842-7 PMC10565972

[32] Falchook G, Infante J, Arkenau HT, Patel MR, Dean E, Borazanci E, et al. First-in-human study of the safety, pharmacokinetics, and pharmacodynamics of first-in-class fatty acid synthase inhibitor TVB-2640 alone and with a taxane in advanced tumors. EClinicalMedicine. 2021;34:100797. 33870151 10.1016/j.eclinm.2021.100797 PMC8040281

[33] Kendall T, Verheij J, Gaudio E, Evert M, Guido M, Goeppert B, et al. Anatomical, histomorphological and molecular classification of cholangiocarcinoma. Liver Int J Int Assoc Study Liver. 2019;39:7–18. 10.1111/liv.14093 30882996

[34] Banales JM, Cardinale V, Carpino G, Marzioni M, Andersen JB, Invernizzi P, et al. Expert consensus document: Cholangiocarcinoma: current knowledge and future perspectives consensus statement from the European Network for the Study of Cholangiocarcinoma (ENS-CCA). Nat Rev Gastroenterol Hepatol. 2016;13:261–80. 27095655 10.1038/nrgastro.2016.51

[35] Corn KC, Windham MA, Rafat M. Lipids in the tumor microenvironment: From cancer progression to treatment. Prog Lipid Res. 2020;80:101055. 32791170 10.1016/j.plipres.2020.101055 PMC7674189

[36] Li L, Che L, Tharp KM, Park HM, Pilo MG, Cao D, et al. Differential requirement for de novo lipogenesis in cholangiocarcinoma and hepatocellular carcinoma of mice and humans. Hepatol Balt Md. 2016;63:1900–13. 10.1002/hep.28508 PMC4874885 26910791

[37] Ruiz de Gauna M, Biancaniello F, González-Romero F, Rodrigues PM, Lapitz A, Gómez-Santos B, et al. Cholangiocarcinoma progression depends on the uptake and metabolization of extracellular lipids. Hepatol Balt Md. 2022;76:1617–33. 10.1002/hep.32344 PMC9790564 35030285

[38] Ko CW, Qu J, Black DD, Tso P. Regulation of intestinal lipid metabolism: current concepts and relevance to disease. Nat Rev Gastroenterol Hepatol. 2020;17:169–83. 32015520 10.1038/s41575-019-0250-7

[39] Benton CR, Han XX, Febbraio M, Graham TE, Bonen A. Inverse relationship between PGC-1alpha protein expression and triacylglycerol accumulation in rodent skeletal muscle. J Appl Physiol Bethesda Md 1985. 2006;100:377–83. 10.1152/japplphysiol.00781.2005 16223979

[40] Hao JW, Wang J, Guo H, Zhao YY, Sun HH, Li YF, et al. CD36 facilitates fatty acid uptake by dynamic palmitoylation-regulated endocytosis. Nat Commun. 2020;11:4765. 32958780 10.1038/s41467-020-18565-8 PMC7505845

[41] Pascual G, Avgustinova A, Mejetta S, Martín M, Castellanos A, Attolini CSO, et al. Targeting metastasis-initiating cells through the fatty acid receptor CD36. Nature. 2017;541:41–5. 27974793 10.1038/nature20791

[42] Yang P, Qin H, Li Y, Xiao A, Zheng E, Zeng H, et al. CD36-mediated metabolic crosstalk between tumor cells and macrophages affects liver metastasis. Nat Commun. 2022;13:5782. 36184646 10.1038/s41467-022-33349-y PMC9527239

[43] Pan J, Fan Z, Wang Z, Dai Q, Xiang Z, Yuan F, et al. CD36 mediates palmitate acid-induced metastasis of gastric cancer via AKT/GSK-3β/β-catenin pathway. J Exp Clin Cancer Res CR. 2019;38:52. 30717785 10.1186/s13046-019-1049-7 PMC6360779

[44] Yu X, Guo C, Fisher PB, Subjeck JR, Wang XY. Scavenger Receptors: Emerging Roles in Cancer Biology and Immunology. Adv Cancer Res. 2015;128:309–64. 26216637 10.1016/bs.acr.2015.04.004 PMC4631385

[45] Zaoui M, Morel M, Ferrand N, Fellahi S, Bastard JP, Lamazière A, et al. Breast-Associated Adipocytes Secretome Induce Fatty Acid Uptake and Invasiveness in Breast Cancer Cells via CD36 Independently of Body Mass Index, Menopausal Status and Mammary Density. Cancers. 2019;11:2012. 31847105 10.3390/cancers11122012 PMC6966437

[46] Feng WW, Wilkins O, Bang S, Ung M, Li J, An J, et al. CD36-Mediated Metabolic Rewiring of Breast Cancer Cells Promotes Resistance to HER2-Targeted Therapies. Cell Rep. 2019;29:3405–.e5. 31825825 10.1016/j.celrep.2019.11.008 PMC6938262

[47] Padthaisong S, Phetcharaburanin J, Klanrit P, Li JV, Namwat N, Khuntikeo N, et al. Integration of global metabolomics and lipidomics approaches reveals the molecular mechanisms and the potential biomarkers for postoperative recurrence in early-stage cholangiocarcinoma. Cancer Metab. 2021;9:30. 34348794 10.1186/s40170-021-00266-5 PMC8335966

[48] Wang J, Li Y. CD36 tango in cancer: signaling pathways and functions. Theranostics. 2019;9:4893–908. 31410189 10.7150/thno.36037 PMC6691380

[49] Wang H, Franco F, Tsui YC, Xie X, Trefny MP, Zappasodi R, et al. CD36-mediated metabolic adaptation supports regulatory T cell survival and function in tumors. Nat Immunol. 2020;21:298–308. 32066953 10.1038/s41590-019-0589-5 PMC7043937

[50] Ma X, Xiao L, Liu L, Ye L, Su P, Bi E, et al. CD36-mediated ferroptosis dampens intratumoral CD8+ T cell effector function and impairs their antitumor ability. Cell Metab. 2021;33:1001–.e5. 33691090 10.1016/j.cmet.2021.02.015 PMC8102368

[51] Black PN, Ahowesso C, Montefusco D, Saini N, DiRusso CC. Fatty Acid Transport Proteins: Targeting FATP2 as a Gatekeeper Involved in the Transport of Exogenous Fatty Acids. MedChemComm. 2016;7:612–22. 27446528 10.1039/C6MD00043F PMC4948302

[52] Acharya R, Shetty SS, Kumari N S. Fatty acid transport proteins (FATPs) in cancer. Chem Phys Lipids. 2023;250:105269. 36462545 10.1016/j.chemphyslip.2022.105269

[53] Dum D, Ocokoljic A, Lennartz M, Hube-Magg C, Reiswich V, Höflmayer D, et al. FABP1 expression in human tumors: a tissue microarray study on 17,071 tumors. Virchows Arch Int J Pathol. 2022;481:945–61. 10.1007/s00428-022-03394-5 PMC9734244 35951102

[54] Baoan Q, Peng L, Jinghan W, Wenchao Z, Nianxin X, Yingxiang Y, et al. Expression and significance of L-FABP in hilar cholangiocarcinoma. Chin J Dig Surg. 2018;17:273–8.

[55] Luis G, Godfroid A, Nishiumi S, Cimino J, Blacher S, Maquoi E, et al. Tumor resistance to ferroptosis driven by Stearoyl-CoA Desaturase-1 (SCD1) in cancer cells and Fatty Acid Biding Protein-4 (FABP4) in tumor microenvironment promote tumor recurrence. Redox Biol. 2021;43:102006. 34030117 10.1016/j.redox.2021.102006 PMC8163990

[56] Zhang Y, Zhang W, Xia M, Xie Z, An F, Zhan Q, et al. High expression of FABP4 in colorectal cancer and its clinical significance. J Zhejiang Univ Sci B. 2021;22:136–45. 33615754 10.1631/jzus.B2000366 PMC7897597

[57] Bussard KM, Mutkus L, Stumpf K, Gomez-Manzano C, Marini FC. Tumor-associated stromal cells as key contributors to the tumor microenvironment. Breast Cancer Res BCR. 2016;18:84. 27515302 10.1186/s13058-016-0740-2 PMC4982339

[58] Nieman KM, Kenny HA, Penicka CV, Ladanyi A, Buell-Gutbrod R, Zillhardt MR, et al. Adipocytes promote ovarian cancer metastasis and provide energy for rapid tumor growth. Nat Med. 2011;17:1498–503. 22037646 10.1038/nm.2492 PMC4157349

[59] Nie J, Zhang J, Wang L, Lu L, Yuan Q, An F, et al. Adipocytes promote cholangiocarcinoma metastasis through fatty acid binding protein 4. J Exp Clin Cancer Res CR. 2017;36:183. 29237483 10.1186/s13046-017-0641-y PMC5729422

[60] Seo N, Kim DY, Choi JY. Cross-Sectional Imaging of Intrahepatic Cholangiocarcinoma: Development, Growth, Spread, and Prognosis. AJR Am J Roentgenol. 2017;209:W64–75. 28570102 10.2214/AJR.16.16923

[61] Chiow SM, Khoo HW, Low JK, Tan CH, Low HM. Imaging mimickers of cholangiocarcinoma: a pictorial review. Abdom Radio N. Y. 2022;47:981–97. 10.1007/s00261-021-03399-9 34978593

[62] Jeong CY, Hah YS, Cho BI, Lee SM, Joo YT, Jung EJ, et al. Fatty acid-binding protein 5 promotes cell proliferation and invasion in human intrahepatic cholangiocarcinoma. Oncol Rep. 2012;28:1283–92. 22825302 10.3892/or.2012.1922

[63] Nakagawa R, Hiep NC, Ouchi H, Sato Y, Harada K. Expression of fatty-acid-binding protein 5 in intrahepatic and extrahepatic cholangiocarcinoma: the possibility of different energy metabolisms in anatomical location. Med Mol Morphol. 2020;53:42–9. 31432248 10.1007/s00795-019-00230-9

[64] Kimura I, Ichimura A, Ohue-Kitano R, Igarashi M. Free Fatty Acid Receptors in Health and Disease. Physiol Rev. 2020;100:171–210. 31487233 10.1152/physrev.00041.2018

[65] Al Mahri S, Malik SS, Al Ibrahim M, Haji E, Dairi G, Mohammad S. Free Fatty Acid Receptors (FFARs) in Adipose: Physiological Role and Therapeutic Outlook. Cells. 2022;11:750. 35203397 10.3390/cells11040750 PMC8870169

[66] Wang X, He S, Gu Y, Wang Q, Chu X, Jin M, et al. Fatty acid receptor GPR120 promotes breast cancer chemoresistance by upregulating ABC transporters expression and fatty acid synthesis. EBioMedicine. 2019;40:251–62. 30738829 10.1016/j.ebiom.2018.12.037 PMC6413582

[67] Moniri NH, Farah Q. Short-chain free-fatty acid G protein-coupled receptors in colon cancer. Biochem Pharm. 2021;186:114483. 33631190 10.1016/j.bcp.2021.114483

[68] Meng FT, Huang M, Shao F, Huang Q. Upregulated FFAR4 correlates with the epithelial-mesenchymal transition and an unfavorable prognosis in human cholangiocarcinoma. Cancer Biomark Sect Dis Markers. 2018;23:353–61. 10.3233/CBM-181358 PMC13078563 30248044

[69] Wu Q, Wang H, Zhao X, Shi Y, Jin M, Wan B, et al. Identification of G-protein-coupled receptor 120 as a tumor-promoting receptor that induces angiogenesis and migration in human colorectal carcinoma. Oncogene. 2013;32:5541–50. 23851494 10.1038/onc.2013.264

[70] Lien EC, Westermark AM, Zhang Y, Yuan C, Li Z, Lau AN, et al. Low glycaemic diets alter lipid metabolism to influence tumour growth. Nature. 2021;599:302–7. 34671163 10.1038/s41586-021-04049-2 PMC8628459

[71] Carracedo A, Cantley LC, Pandolfi PP. Cancer metabolism: fatty acid oxidation in the limelight. Nat Rev Cancer. 2013;13:227–32. 23446547 10.1038/nrc3483 PMC3766957

[72] Ma Y, Temkin SM, Hawkridge AM, Guo C, Wang W, Wang XY, et al. Fatty acid oxidation: An emerging facet of metabolic transformation in cancer. Cancer Lett. 2018;435:92–100. 30102953 10.1016/j.canlet.2018.08.006 PMC6240910

[73] Hoy AJ, Nagarajan SR, Butler LM. Tumour fatty acid metabolism in the context of therapy resistance and obesity. Nat Rev Cancer. 2021;21:753–66. 34417571 10.1038/s41568-021-00388-4

[74] Ju HQ, Lin JF, Tian T, Xie D, Xu RH. NADPH homeostasis in cancer: functions, mechanisms and therapeutic implications. Signal Transduct Target Ther. 2020;5:231. 33028807 10.1038/s41392-020-00326-0 PMC7542157

[75] Lee CK, Jeong SH, Jang C, Bae H, Kim YH, Park I, et al. Tumor metastasis to lymph nodes requires YAP-dependent metabolic adaptation. Science. 2019;363:644–9. 30733421 10.1126/science.aav0173

[76] Hubbard GK, Mutton LN, Khalili M, McMullin RP, Hicks JL, Bianchi-Frias D, et al. Combined MYC Activation and Pten Loss Are Sufficient to Create Genomic Instability and Lethal Metastatic Prostate Cancer. Cancer Res. 2016;76:283–92. 26554830 10.1158/0008-5472.CAN-14-3280 PMC5006678

[77] Joshi M, Kim J, D’Alessandro A, Monk E, Bruce K, Elajaili H, et al. CPT1A Over-Expression Increases Reactive Oxygen Species in the Mitochondria and Promotes Antioxidant Defenses in Prostate Cancer. Cancers. 2020;12:3431. 33218188 10.3390/cancers12113431 PMC7709014

[78] Wong BW, Wang X, Zecchin A, Thienpont B, Cornelissen I, Kalucka J, et al. The role of fatty acid β-oxidation in lymphangiogenesis. Nature. 2017;542:49–54. 28024299 10.1038/nature21028

[79] Hassannia B, Vandenabeele P, Vanden Berghe T. Targeting Ferroptosis to Iron Out Cancer. Cancer Cell. 2019;35:830–49. 31105042 10.1016/j.ccell.2019.04.002

[80] Hayes JD, Dinkova-Kostova AT, Tew KD. Oxidative Stress in Cancer. Cancer Cell. 2020;38:167–97. 32649885 10.1016/j.ccell.2020.06.001 PMC7439808

[81] Dodson M, Castro-Portuguez R, Zhang DD. NRF2 plays a critical role in mitigating lipid peroxidation and ferroptosis. Redox Biol. 2019;23:101107. 30692038 10.1016/j.redox.2019.101107 PMC6859567

[82] Maeng S, Lee HW, Bashir Q, Kim TI, Hong SJ, Lee TJ, et al. Oxidative stress-mediated mouse liver lesions caused by Clonorchis sinensis infection. Int J Parasitol. 2016;46:195–204. 26718397 10.1016/j.ijpara.2015.11.003

[83] Dechakhamphu S, Pinlaor S, Sitthithaworn P, Nair J, Bartsch H, Yongvanit P. Lipid peroxidation and etheno DNA adducts in white blood cells of liver fluke-infected patients: protection by plasma alpha-tocopherol and praziquantel. Cancer Epidemiol Biomark Prev Publ Am Assoc Cancer Res Cosponsored Am Soc Prev Oncol. 2010;19:310–8. 10.1158/1055-9965.EPI-09-0849 20056652

[84] Jiang X, Stockwell BR, Conrad M. Ferroptosis: mechanisms, biology and role in disease. Nat Rev Mol Cell Biol. 2021;22:266–82. 33495651 10.1038/s41580-020-00324-8 PMC8142022

[85] Fu S, Zhang Q, Zhang C. Research Update for Ferroptosis and Cholangiocarcinoma. Crit Rev Oncol Hematol. 2024 Apr;104356. 10.1016/j.critrevonc.2024.104356 38641134

[86] Raggi C, Gammella E, Correnti M, Buratti P, Forti E, Andersen JB, et al. Dysregulation of Iron Metabolism in Cholangiocarcinoma Stem-like Cells. Sci Rep. 2017;7:17667. 29247214 10.1038/s41598-017-17804-1 PMC5732280

[87] Lei S, Cao W, Zeng Z, Zhang Z, Jin B, Tian Q, et al. JUND/linc00976 promotes cholangiocarcinoma progression and metastasis, inhibits ferroptosis by regulating the miR-3202/GPX4 axis. Cell Death Dis. 2022;13:967. 36400758 10.1038/s41419-022-05412-5 PMC9674662

[88] Zeng C, Lin J, Zhang K, Ou H, Shen K, Liu Q, et al. SHARPIN promotes cell proliferation of cholangiocarcinoma and inhibits ferroptosis via p53/SLC7A11/GPX4 signaling. Cancer Sci. 2022;113:3766–75. 35968603 10.1111/cas.15531 PMC9633309

[89] Mortensen MS, Ruiz J, Watts JL. Polyunsaturated Fatty Acids Drive Lipid Peroxidation during Ferroptosis. Cells. 2023;12:804. 36899940 10.3390/cells12050804 PMC10001165

[90] Chen X, Kang R, Kroemer G, Tang D. Broadening horizons: the role of ferroptosis in cancer. Nat Rev Clin Oncol. 2021;18:280–96. 33514910 10.1038/s41571-020-00462-0

[91] Sánchez-Martínez R, Cruz-Gil S, García-Álvarez MS, Reglero G, Ramírez de Molina A. Complementary ACSL isoforms contribute to a non-Warburg advantageous energetic status characterizing invasive colon cancer cells. Sci Rep. 2017;7:11143. 28894242 10.1038/s41598-017-11612-3 PMC5593891

[92] Chen J, Ding C, Chen Y, Hu W, Lu Y, Wu W, et al. ACSL4 promotes hepatocellular carcinoma progression via c-Myc stability mediated by ERK/FBW7/c-Myc axis. Oncogenesis. 2020;9:42. 32350243 10.1038/s41389-020-0226-z PMC7190855

[93] Zhang Y, Li S, Li F, Lv C, Yang QK. High-fat diet impairs ferroptosis and promotes cancer invasiveness via downregulating tumor suppressor ACSL4 in lung adenocarcinoma. Biol Direct. 2021;16:10. 34053456 10.1186/s13062-021-00294-7 PMC8166005

[94] Kwon YS, Lee MG, Baek J, Kim NY, Jang H, Kim S. Acyl-CoA synthetase-4 mediates radioresistance of breast cancer cells by regulating FOXM1. Biochem Pharm. 2021;192:114718. 34358518 10.1016/j.bcp.2021.114718

[95] Liu S, Fan S, Wang Y, Chen R, Wang Z, Zhang Y, et al. ACSL4 serves as a novel prognostic biomarker correlated with immune infiltration in Cholangiocarcinoma. BMC Cancer. 2023;23:444. 37193981 10.1186/s12885-023-10903-5 PMC10186676

[96] Song Y, Zhang Z, Chai Q, Zheng H, Qi Y, Xia G, et al. Quercetin Inhibits Intrahepatic Cholangiocarcinoma by Inducing Ferroptosis and Inhibiting Invasion via the NF-[Formula: see text]B Pathway. Am J Chin Med. 2023;51:701–21. 36823098 10.1142/S0192415X23500337

[97] Currie E, Schulze A, Zechner R, Walther TC, Farese RV. Cellular fatty acid metabolism and cancer. Cell Metab. 2013;18:153–61. 23791484 10.1016/j.cmet.2013.05.017 PMC3742569

[98] Mansini AP, Peixoto E, Thelen KM, Gaspari C, Jin S, Gradilone SA. The cholangiocyte primary cilium in health and disease. Biochim Biophys Acta Mol Basis Dis. 2018;1864:1245–53. 28625917 10.1016/j.bbadis.2017.06.006 PMC5732091

[99] Pant K, Richard S, Gradilone SA. Short-Chain Fatty Acid Butyrate Induces Cilia Formation and Potentiates the Effects of HDAC6 Inhibitors in Cholangiocarcinoma Cells. Front Cell Dev Biol. 2021;9:809382. 35096835 10.3389/fcell.2021.809382 PMC8793355

[100] Mansini AP, Lorenzo Pisarello MJ, Thelen KM, Cruz-Reyes M, Peixoto E, Jin S, et al. MicroRNA (miR)-433 and miR-22 dysregulations induce histone-deacetylase-6 overexpression and ciliary loss in cholangiocarcinoma. Hepatol Balt Md. 2018;68:561–73. 10.1002/hep.29832 PMC6078832 29406621

[101] Pascual G, Domínguez D, Elosúa-Bayes M, Beckedorff F, Laudanna C, Bigas C, et al. Dietary palmitic acid promotes a prometastatic memory via Schwann cells. Nature. 2021;599:485–90. 34759321 10.1038/s41586-021-04075-0

[102] Xiao Q, Lan Z, Zhang S, Ren H, Wang S, Wang P, et al. Overexpression of ZNF488 supports pancreatic cancer cell proliferation and tumorigenesis through inhibition of ferroptosis via regulating SCD1-mediated unsaturated fatty acid metabolism. Biol Direct. 2023;18:77. 37986084 10.1186/s13062-023-00421-6 PMC10658979

[103] Zhang J, Song F, Zhao X, Jiang H, Wu X, Wang B, et al. EGFR modulates monounsaturated fatty acid synthesis through phosphorylation of SCD1 in lung cancer. Mol Cancer. 2017;16:127. 28724430 10.1186/s12943-017-0704-x PMC5518108

[104] Gleba JJ, Marlow LA, Miller EE, Miller JL, Alasonyalilar-Demirer A, Guo Y, et al. Abstract 1004: Defining stearoyl-CoA desaturase 1 as a molecular therapeutic target against cholangiocarcinoma. Cancer Res. 2021;81:1004–1004.

[105] Kado T, Kusakari N, Tamaki T, Murota K, Tsujiuchi T, Fukushima N. Oleic acid stimulates cell proliferation and BRD4-L-MYC-dependent glucose transporter transcription through PPARα activation in ovarian cancer cells. Biochem Biophys Res Commun. 2023;657:24–34. 36965420 10.1016/j.bbrc.2023.03.051

[106] Zhang H, Zhu K, Zhang R, Guo Y, Wang J, Liu C, et al. Oleic acid-PPARγ-FABP4 loop fuels cholangiocarcinoma colonization in lymph node metastases microenvironment. Hepatol Baltim Md. 2024 Feb; 10.1097/HEP.0000000000000784 38377465

[107] Ding DY, Gan XJ, Zhang JN, Hou GJ, Tao QF, Sun DP, et al. Serum thrombospondin-1 serves as a novel biomarker and agonist of gemcitabine-based chemotherapy in intrahepatic cholangiocarcinoma. Br J Cancer. 2023;128:907–17. 36526676 10.1038/s41416-022-02101-0 PMC9977883

[108] Gorjao R, Dos Santos CMM, Serdan TDA, Diniz VLS, Alba-Loureiro TC, Cury-Boaventura MF, et al. New insights on the regulation of cancer cachexia by N-3 polyunsaturated fatty acids. Pharm Ther. 2019;196:117–34. 10.1016/j.pharmthera.2018.12.001 30521881

[109] Lin CR, Chu TM, Luo A, Huang SJ, Chou HY, Lu MW, et al. Omega-3 polyunsaturated fatty acids suppress metastatic features of human cholangiocarcinoma cells by suppressing twist. J Nutr Biochem. 2019;74:108245. 31678746 10.1016/j.jnutbio.2019.108245

[110] Lim K, Han C, Xu L, Isse K, Demetris AJ, Wu T. Cyclooxygenase-2-derived prostaglandin E2 activates beta-catenin in human cholangiocarcinoma cells: evidence for inhibition of these signaling pathways by omega 3 polyunsaturated fatty acids. Cancer Res. 2008;68:553–60. 18199552 10.1158/0008-5472.CAN-07-2295

[111] Haitao J, Wei Z, Lian’gang M, Yunjie C, Jingyu C. The effect of docosahexaenoic acid combined with cyclooxygenase-2 selective inhibitor NS-398 on the apoptosis of bile duct cancer cells QBC939. Chin J Hepatobiliary Surg. 2018;24:336–40.

[112] Hayashi N, Yamamoto H, Hiraoka N, Dono K, Ito Y, Okami J, et al. Differential expression of cyclooxygenase-2 (COX-2) in human bile duct epithelial cells and bile duct neoplasm. Hepatol Balt Md. 2001;34:638–50. 10.1053/jhep.2001.28198 11584358

[113] Yao L, Han C, Song K, Zhang J, Lim K, Wu T. Omega-3 Polyunsaturated Fatty Acids Upregulate 15-PGDH Expression in Cholangiocarcinoma Cells by Inhibiting miR-26a/b Expression. Cancer Res. 2015;75:1388–98. 25691459 10.1158/0008-5472.CAN-14-2561 PMC4383692

[114] Gül-Utku Ö, Karatay E, Ergül B, Kisa Ü, Erdin Z, Oğuz D. The Role of Resolvin D1 in the Differential Diagnosis of the Cholangiocarcinoma and Benign Biliary Diseases. Clin Lab. 2020 May;66. 10.7754/Clin.Lab.2020.200212 32390401

[115] Khophai S, Thanee M, Techasen A, Namwat N, Klanrit P, Titapun A, et al. Zileuton suppresses cholangiocarcinoma cell proliferation and migration through inhibition of the Akt signaling pathway. OncoTargets Ther. 2018;11:7019–29. 10.2147/OTT.S178942 PMC6198876 30410359

[116] Shen X, Shen X. A potential role for aspirin in the prevention and treatment of cholangiocarcinoma. Int J Cancer. 2021;148:1323–30. 32997790 10.1002/ijc.33323

[117] Wu T, Han C, Lunz JG, Michalopoulos G, Shelhamer JH, Demetris AJ. Involvement of 85-kd cytosolic phospholipase A(2) and cyclooxygenase-2 in the proliferation of human cholangiocarcinoma cells. Hepatol Balt Md. 2002;36:363–73. 10.1053/jhep.2002.34743 12143044

[118] Du M, Shi F, Zhang H, Xia S, Zhang M, Ma J, et al. Prostaglandin E2 promotes human cholangiocarcinoma cell proliferation, migration and invasion through the upregulation of β-catenin expression via EP3-4 receptor. Oncol Rep. 2015;34:715–26. 26058972 10.3892/or.2015.4043

[119] Sun B, Rong R, Jiang H, Zhang H, Wang Y, Bai X, et al. Prostaglandin E2 receptor EP1 phosphorylate CREB and mediates MMP2 expression in human cholangiocarcinoma cells. Mol Cell Biochem. 2013;378:195–203. 23494562 10.1007/s11010-013-1610-1

[120] Jongthawin J, Techasen A, Loilome W, Yongvanit P, Namwat N. Anti-inflammatory agents suppress the prostaglandin E2 production and migration ability of cholangiocarcinoma cell lines. Asian Pac J Cancer Prev APJCP. 2012;47–51. 23480764

[121] Lu L, Byrnes K, Han C, Wang Y, Wu T. miR-21 targets 15-PGDH and promotes cholangiocarcinoma growth. Mol Cancer Res MCR. 2014;12:890–900. 24699315 10.1158/1541-7786.MCR-13-0419 PMC4058387

[122] Qiu J, Zhao Z, Suo H, Paraghamian SE, Hawkins GM, Sun W, et al. Linoleic acid exhibits anti-proliferative and anti-invasive activities in endometrial cancer cells and a transgenic model of endometrial cancer. Cancer Biol Ther. 2024;25:2325130. 38465855 10.1080/15384047.2024.2325130 PMC10936646

[123] Serna-Marquez N, Diaz-Aragon R, Reyes-Uribe E, Cortes-Reynosa P, Salazar EP. Linoleic acid induces migration and invasion through FFAR4- and PI3K-/Akt-dependent pathway in MDA-MB-231 breast cancer cells. Med Oncol Northwood Lond Engl. 2017;34:111. 10.1007/s12032-017-0969-3 28456993

[124] Li J, Lu J, Lv S, Sun S, Liu C, Xu F, et al. Linoleic acid pathway disturbance contributing to potential cancerization of intrahepatic bile duct stones into intrahepatic cholangiocarcinoma. BMC Gastroenterol. 2022;22:269. 35637430 10.1186/s12876-022-02354-2 PMC9153149

[125] Chen R, Zheng D, Li Q, Xu S, Ye C, Jiang Q, et al. Immunotherapy of cholangiocarcinoma: Therapeutic strategies and predictive biomarkers. Cancer Lett. 2022;546:215853. 35921970 10.1016/j.canlet.2022.215853

[126] Nava Lauson CB, Tiberti S, Corsetto PA, Conte F, Tyagi P, Machwirth M, et al. Linoleic acid potentiates CD8+ T cell metabolic fitness and antitumor immunity. Cell Metab. 2023;35:633.e9. 36898381 10.1016/j.cmet.2023.02.013

[127] den Hartigh LJ. Conjugated Linoleic Acid Effects on Cancer, Obesity, and Atherosclerosis: A Review of Pre-Clinical and Human Trials with Current Perspectives. Nutrients. 2019;11:370. 30754681 10.3390/nu11020370 PMC6413010

[128] Liput KP, Lepczyński A, Ogłuszka M, Nawrocka A, Poławska E, Grzesiak A, et al. Effects of Dietary n-3 and n-6 Polyunsaturated Fatty Acids in Inflammation and Cancerogenesis. Int J Mol Sci. 2021;22:6965. 34203461 10.3390/ijms22136965 PMC8268933

[129] DiNicolantonio JJ, O’Keefe J. The Importance of Maintaining a Low Omega-6/Omega-3 Ratio for Reducing the Risk of Autoimmune Diseases, Asthma, and Allergies. Mo Med. 2021;118:453–9. 34658440 PMC8504498

[130] Sousa TMde, Santos LCD. Dietary fatty acids, omega-6/omega-3 ratio and cholesterol intake associated with depressive symptoms in low-risk pregnancy. Nutr Neurosci. 2022;25:642–7. 32654629 10.1080/1028415X.2020.1792618

[131] DiNicolantonio JJ, O’Keefe JH. Importance of maintaining a low omega-6/omega-3 ratio for reducing inflammation. Open Heart. 2018;5:e000946. 30564378 10.1136/openhrt-2018-000946 PMC6269634

[132] Dierge E, Debock E, Guilbaud C, Corbet C, Mignolet E, Mignard L, et al. Peroxidation of n-3 and n-6 polyunsaturated fatty acids in the acidic tumor environment leads to ferroptosis-mediated anticancer effects. Cell Metab. 2021;33:1701–.e5. 34118189 10.1016/j.cmet.2021.05.016

[133] Campos-Perez W, Perez-Robles M, Rodriguez-Echevarria R, Rivera-Valdés JJ, Rodríguez-Navarro FM, Rivera-Leon EA, et al. High dietary ω-6:ω-3 PUFA ratio and simple carbohydrates as a potential risk factors for gallstone disease: A cross-sectional study. Clin Res Hepatol Gastroenterol. 2022;46:101802. 34896648 10.1016/j.clinre.2021.101802

[134] Hatzivassiliou G, Zhao F, Bauer DE, Andreadis C, Shaw AN, Dhanak D, et al. ATP citrate lyase inhibition can suppress tumor cell growth. Cancer Cell. 2005;8:311–21. 16226706 10.1016/j.ccr.2005.09.008

[135] Liu J, Luo X, Guo R, Jing W, Lu H. Cell Metabolomics Reveals Berberine-Inhibited Pancreatic Cancer Cell Viability and Metastasis by Regulating Citrate Metabolism. J Proteome Res. 2020;19:3825–36. 32692565 10.1021/acs.jproteome.0c00394

[136] Liang JJ, Zhou XF, Long H, Li CY, Wei J, Yu XQ, et al. Recent advance of ATP citrate lyase inhibitors for the treatment of cancer and related diseases. Bioorg Chem. 2024;142:106933. 37890210 10.1016/j.bioorg.2023.106933

[137] He Y, Sun MM, Zhang GG, Yang J, Chen KS, Xu WW, et al. Targeting PI3K/Akt signal transduction for cancer therapy. Signal Transduct Target Ther. 2021;6:425. 34916492 10.1038/s41392-021-00828-5 PMC8677728

[138] Zhao Q, Lin X, Wang G. Targeting SREBP-1-Mediated Lipogenesis as Potential Strategies for Cancer. Front Oncol. 2022;12:952371. 35912181 10.3389/fonc.2022.952371 PMC9330218

[139] Zhou X, Li Y, Yang C, Chen D, Wang T, Liu T, et al. Cordycepin reprogramming lipid metabolism to block metastasis and EMT via ERO1A/mTOR/SREBP1 axis in cholangiocarcinoma. Life Sci. 2023;327:121698. 37080351 10.1016/j.lfs.2023.121698

[140] Ye Z, Zhuo Q, Hu Q, Xu X, Mengqi L, Zhang Z, et al. FBW7-NRA41-SCD1 axis synchronously regulates apoptosis and ferroptosis in pancreatic cancer cells. Redox Biol. 2021;38:101807. 33271455 10.1016/j.redox.2020.101807 PMC7710650

[141] Lei G, Mao C, Yan Y, Zhuang L, Gan B. Ferroptosis, radiotherapy, and combination therapeutic strategies. Protein Cell. 2021;12:836–57. 33891303 10.1007/s13238-021-00841-y PMC8563889

